# Separation from mechanical ventilation and survival after spinal cord injury: a systematic review and meta-analysis

**DOI:** 10.1186/s13613-021-00938-x

**Published:** 2021-10-24

**Authors:** Annia F. Schreiber, Jacopo Garlasco, Fernando Vieira, Yie Hui Lau, Dekel Stavi, David Lightfoot, Andrea Rigamonti, Karen Burns, Jan O. Friedrich, Jeffrey M. Singh, Laurent J. Brochard

**Affiliations:** 1 Interdepartmental Division of Critical Care Medicine, University of Toronto, Toronto, Canada; 2 Keenan Research Centre, Li Ka Shing Knowledge Institute, Unity Health Toronto, St. Michael’s Hospital, Toronto, Canada; 3 Department of Public Health Sciences and Pediatrics, University of Turin, Turin, Italy; 4 Department of Anaesthesiology, Intensive Care and Pain Medicine, Tan Tock Seng Hospital, Singapore, Singapore; 5 Health Sciences Library, Unity Health Toronto, St. Michael’s Hospital, Toronto, Canada; 6 Department of Medicine, University Health Network, Toronto, Canada

**Keywords:** Spinal cord injury, Mechanical ventilation, Respiratory failure, Weaning, Intensive care unit, Rehabilitation

## Abstract

**Background:**

Prolonged need for mechanical ventilation greatly impacts life expectancy of patients after spinal cord injury (SCI). Weaning outcomes have never been systematically assessed. In this systematic review and meta-analysis, we aimed to investigate the probability of weaning success, duration of mechanical ventilation, mortality, and their predictors in mechanically ventilated patients with SCI.

**Methods:**

We searched six databases from inception until August 2021 for randomized-controlled trials and observational studies enrolling adult patients (≥ 16 years) with SCI from any cause requiring mechanical ventilation. Titles and abstracts were screened independently by two reviewers. Full texts of the identified articles were then assessed for eligibility. Data were extracted independently and in duplicate by pairs of authors, using a standardized data collection form. Synthetic results are reported as meta-analytic means and proportions, based on random effects models.

**Results:**

Thirty-nine studies (14,637 patients, mean age 43) were selected. Cervical lesions were predominant (12,717 patients had cervical lesions only, 1843 in association with other levels’ lesions). Twenty-five studies were conducted in intensive care units (ICUs), 14 in rehabilitative settings.

In ICU, the mean time from injury to hospitalization was 8 h [95% CI 7–9], mean duration of mechanical ventilation 27 days [20–34], probability of weaning success 63% [45–78] and mortality 8% [5–11]. Patients hospitalized in rehabilitation centres had a greater number of high-level lesions (C3 or above), were at 40 days [29–51] from injury and were ventilated for a mean of 97 days [65–128]; 82% [70–90] of them were successfully weaned, while mortality was 1% [0–19].

**Conclusions:**

Although our study highlights the lack of uniform definition of weaning success, of clear factors associated with weaning outcomes, and of high-level evidence to guide optimal weaning in patients with SCI, it shows that around two-thirds of mechanically ventilated patients can be weaned in ICU after SCI. A substantial gain in weaning success can be obtained during rehabilitation, with additional duration of stay but minimal increase in mortality. The study is registered with PROSPERO (CRD42020156788).

**Supplementary Information:**

The online version contains supplementary material available at 10.1186/s13613-021-00938-x.

## Background

Spinal cord injury (SCI) is a dramatic and life-changing event. The prevalence of traumatic SCI was reported to be between 236 and 4187 (with a most likely estimate around 500) cases per million worldwide[1, 2]. More than 75% of these traumatic injuries occur in subjects under 45 years of age, the majority of which being between the age of 16 and 30 [3, 4]. When the origin is non-traumatic, the most common causes are age-related conditions, including tumors, vascular/inflammatory diseases, and degenerative affections [5, 6].

It is estimated that more than 90% of all traumatic cervical SCIs require intubation, a majority of them require a tracheostomy, and up to 40% of the patients with complete cervical lesions remain ventilator-dependent [7, 8]. Despite many advances in the management of patients with SCI, mortality for individuals who are ventilator-dependent remains very high [9, 10]. Life expectancy largely depends on the possibility to wean them off the ventilator, as do health care expenses and quality of life [4, 11, 12].

Despite the crucial role of mechanical ventilation, studies looking at specific approaches to weaning from mechanical ventilation in SCI are scarce and published research on the topic mainly consists of retrospective observational studies and small case series [13]. The existing guidelines on respiratory management after SCI are mainly focused on non-critically ill patients [14, 15]. The probability of weaning success remains difficult to predict; no previous systematic review or meta-analysis has been conducted on the topic, and no societal guidelines or recommendations on weaning are available for this population [16].

Our aim was to investigate the probability of weaning success, duration of mechanical ventilation, mortality, and their predictors in mechanically ventilated adult patients with SCI.

## Methods

We conducted this review in accordance with the Preferred Reporting Items for Systematic Reviews and Meta-Analyses [17]. The protocol was registered in the International Prospective Register of Systematic Reviews (PROSPERO; CRD42020156788). Ethics approval was not required because all study data had been previously published and, in this study, we did not include any individual patient data.

### Eligibility criteria, literature search, study selection

We conducted a systematic literature search from inception to August 2021 to identify studies enrolling adult patients (≥ 16 years of age) with SCI (at any level and from any cause, including traumatic and non-traumatic) who required mechanical ventilation, and evaluating at least one of the 3 following key outcomes: probability of weaning success, duration of mechanical ventilation, or mortality. Studies enrolling both ventilated and non-ventilated subjects were included if characteristics and outcomes of ventilated subjects could be extracted separately, or if non-ventilated subjects represented a minority (< 20%) of the study population. Case reports and case series with fewer than 10 participants were excluded. Studies enrolling > 20% of subjects under 16 years of age whose outcomes were not separable from the remaining study population were also excluded, according to usual recommendations [18].

The following six electronic bibliographic databases were searched using a comprehensive search strategy developed by one of the authors (DL), an information specialist who has expertise in database searching: OVID Medline, CINAHL, the Cochrane Central Register of Controlled Trials and the Cochrane Database of Systematic Reviews, Ovid Embase and Scopus (Additional file 1: Appendix S1).

### Outcomes

The primary outcome was the probability of weaning success (complete and partial liberation from the ventilator). The secondary outcome was either time to reach liberation from the ventilator or duration of mechanical ventilation, according to each study analysis. Additional secondary outcomes included mortality at any time point, intensive care unit (ICU) or rehabilitation unit lengths of stay (according to each study setting), hospital length of stay, and probability of tracheostomy and decannulation. Factors associated with the probability of weaning-related outcomes (weaning success and duration of mechanical ventilation) were also assessed.

Studies conducted in ICUs and studies conducted in rehabilitation units were analyzed separately, given the differences in these study settings.

### Data extraction and quality assessment

Each title and abstract were screened independently by two reviewers. Full texts of all the articles identified as relevant by either reviewer were then assessed for eligibility. Pairs of authors, independently and in duplicate, reviewed and selected studies meeting inclusion criteria and extracted data using a standardized data collection form (Additional file 2: Appendix S2). Consensus on extracted data was reached by discussion, and disagreements were resolved by arbitration with a third author. The same authors evaluated the quality of included studies using the Newcastle–Ottawa Scale [19, 20].

### Statistical analysis

The complete statistical analysis is detailed in the Supplemental Digital Content.

For each study mean values and standard deviations (SDs) were preferred where available. In their absence, available measures including medians, ranges and 1st–3rd quartile intervals (Q1-Q3) were used to estimate sample means and SDs [21, 22]. For studies reporting values for subgroups only, the overall estimates were computed according to the Cochrane handbook guidelines [23].

The R package “meta” was used for all meta-analytic computations and plotting [24]. Synthetic results are reported as meta-analytic means for continuous variables, and both as crude proportions and meta-analytic proportions, for categorical variables. To incorporate between-study heterogeneity [25], all analyses were performed using random effects models [26].

A 95% study confidence interval (95% CI) was evaluated for all characteristics and outcomes through the standard normal distribution for meta-analytic means and according to the Clopper–Pearson method for meta-proportions [27]. Study weights were generated using the inverse variance method. Heterogeneity among studies was assessed by visual inspection of the forest plots, using the I^2^ statistic (threshold level for significant heterogeneity: ≥ 50%), Cochran’s Q and the Chi-squared test for homogeneity (significance level for heterogeneity: *p* < 0.1)[28].

Predefined subgroup analysis (significance level α = 0.05) was performed dividing studies conducted in ICUs vs rehabilitative settings.

All statistical analyses were performed using R (version 4.0.3) [29].

## Results

### Literature search and study characteristics

**Fig. 1:**
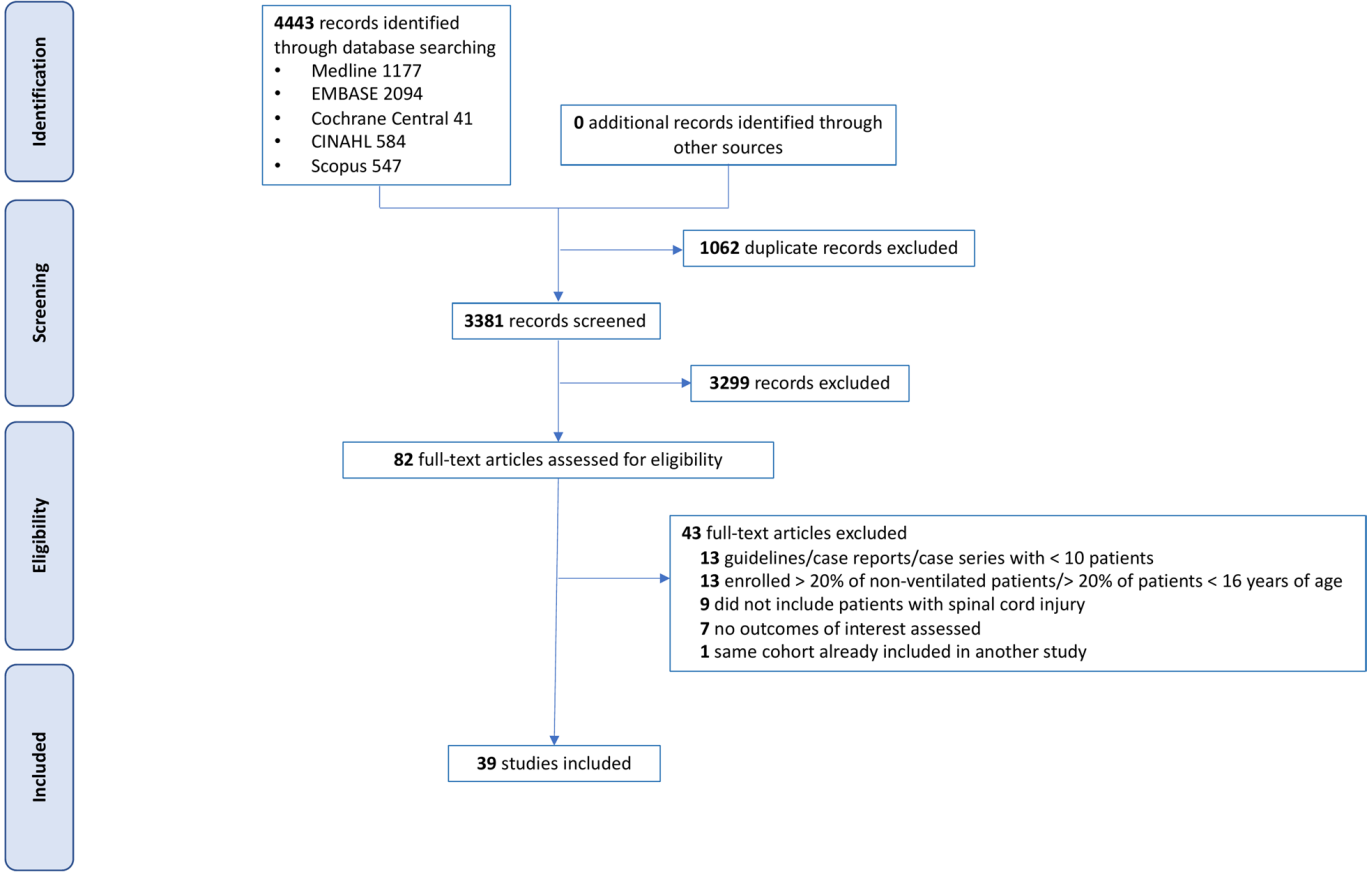
Flowchart of the study selection process according to PRISMA (www.prisma-statement.org)

The search strategy identified 4443 records that were screened for eligibility. Eighty-two full-text articles were assessed and 43 were excluded (Fig. 1). Thirty-nine studies were finally included in the systematic review.

The characteristics of the studies are reported in Additional file 1: Tables S1 and S2. The majority of the studies (26, 67%) were published in SCI or trauma journals, 7 (18%) in surgical journals, 4 (10%) in rehabilitative and 3 (8%) in critical care/general medicine journals. Six studies were multicentric [30–35], 33 were conducted at a single site [8, 12, 36–66]. Most studies were conducted either in Level I [8, 37, 42, 46, 57, 61, 63, 66] or in unspecified trauma/SCI treatment centers [12, 31, 35, 36, 38, 39, 41, 43, 47, 48, 50–52, 55, 59, 62, 65]. Twenty-five studies were run in ICUs [12, 30–33, 35–44, 46, 57, 59–62, 64–66], 14 in rehabilitative settings [34, 45, 47–56, 58, 61] (Additional file 1: Table S1). In 8 studies the cause of the injury was not specified [33, 43, 47, 49, 51, 53, 54, 58], all the remaining studies enrolled traumatic SCI patients, 2 of them [55, 61] enrolled both traumatic and a few non-traumatic patients, whose outcomes were not separable. Among the studies focusing on traumatic SCI, 6 excluded patients with concomitant traumatic brain injury [30, 32, 40, 60, 62, 66], 1 excluded patients with multiple spinal cord lesions [65] and 1 excluded patients with concomitant extra-spine lesions [33].

Quality of included studies is reported in Additional file 1: Table S3.

A total of 14,637 patients were enrolled (13,763 in ICU, 874 in rehabilitation units), 80% of them were male and their mean age was 43 years [95% CI 41–45]. The mean time from injury to hospitalization was 8 h [95% CI 7–9] for studies conducted in ICU, 40 days [95% CI 29–51] for studies performed in rehabilitative units (Additional file 1: Tables S1, S4). Compared to patients hospitalized in ICU, patients admitted to rehabilitation units were more frequently male (84% vs 79%, *p* = 0.029), had more assault lesions (11% vs 1%, *p* < 0.0001), and more high-level (C3 or above compared to C4 or below) lesions (55% vs 14%, *p* = 0.0003). Other baseline characteristics of patients enrolled in each ICU or rehabilitative study are summarized in Additional file 1: Table S4.

### Primary outcome: probability of weaning from mechanical ventilation after SCI

**Fig. 2:**
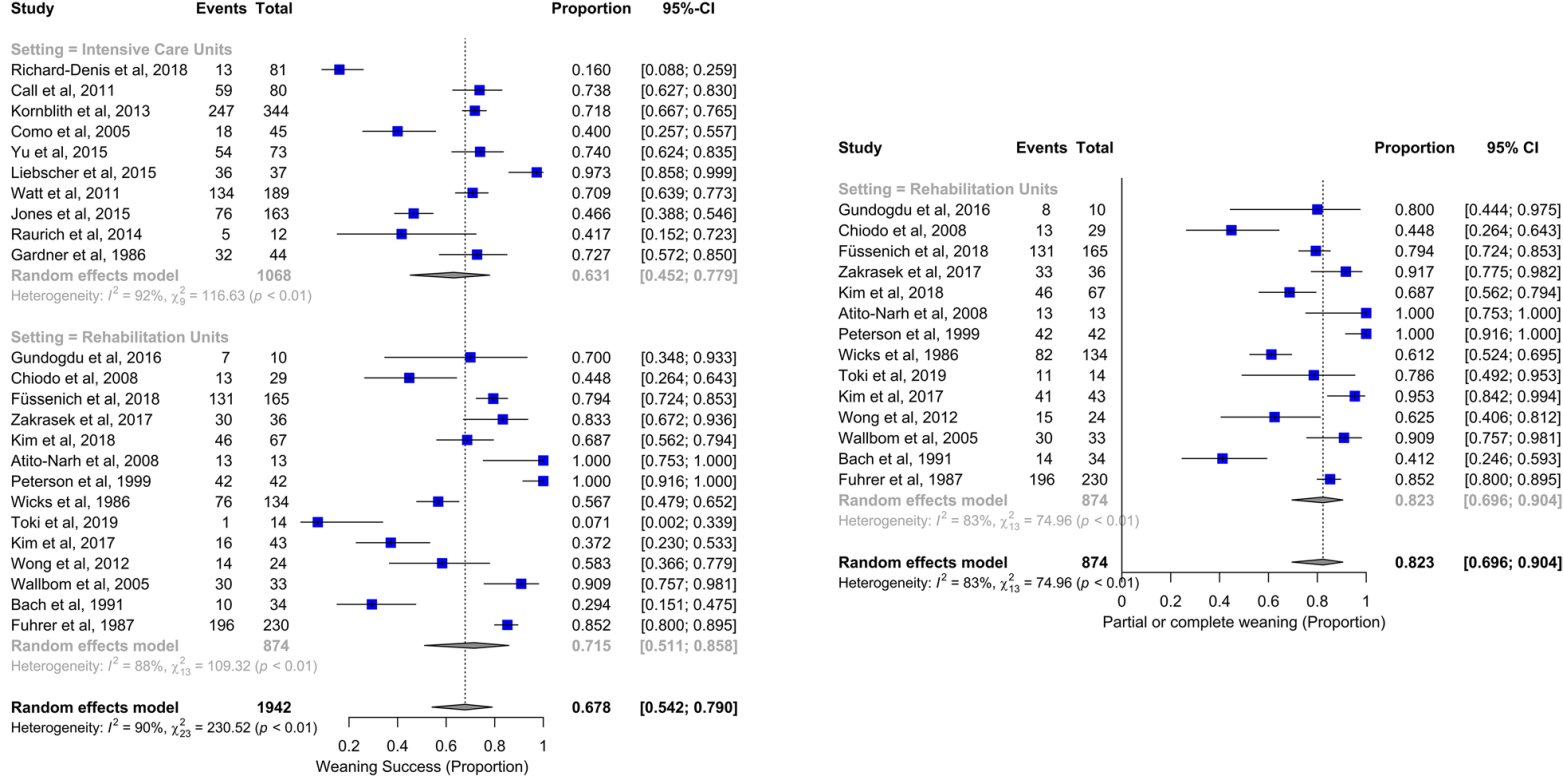
Forest plots for the outcome of complete liberation from the ventilator (left panel) and for the outcome of partial or complete weaning after rehabilitation (right panel). Studies are presented according to setting classification (intensive care units vs rehabilitation units): both overall and subgroup estimates are reported. Number of events and total number of patients at risk are reported for each study. The computation process has been described in the Methods. CI: confidence interval

Twenty-four studies (10 conducted in ICU and all the 14 studies conducted in rehabilitative wards) assessed the probability of complete liberation, and 6 the probability of partial liberation from the ventilator (Additional file 1: Table S2). The crude and meta-analytic proportions of complete and partial weaning are reported in Table 1 and Fig. 2. 63% [45–78%] of the patients hospitalized in ICU were completely separated from the ventilator; 72% [51–86%] of the patients admitted to a rehabilitative ward were completely, and 82% [70–90%] were either completely or partially liberated from the ventilator.

**Table 1:** Main outcomes after injury

Outcomes after injury	Means and proportions	Overall (*n* = 14,637)	ICU (*n* = 13,763)	Rehab unit (*n* = 874)	Number of studies	Subgroup differences	
						p-value^#^	*p*-value^#^ (excluding Branco et al. and Anand et al.)
Weaning success, n (%)	Crude	1299/1942 (66.9%)	674/1068 (63.1%)	625/874 (71.5%)	24 (10 + 14)		
	Meta-analytic	67.8% [54.2–79%]	63.1% [45.2–77.9%]	71.5% [51.1–85.8%]		0.5059	–
Partial weaning, n (%)	Crude	75/195 (38.5%)	2/44 (4.5%)	73/151 (48.3%)	6 (1 + 5)		
	Meta-analytic	31.9% [7.4–73.3%]	4.5% [1.1–16.4%]	43.3% [11.1–82.4%]		0.0582	–
Partial or total weaning, n (%)	Crude	1351/1942 (69.6%)	676/1068 (63.3%)	675/874 (77.2%)	24 (10 + 14)		
	Meta-analytic	75.4% [63.9–84.1%]	63.7% [45.6–78.6%]	82.3% [69.6–90.4%]		0.0610	–
Tracheostomy, n (%)	Crude	8344/13371 (62.4%)*	8142/13165 (61.8%)*	202/206 (98.1%)	27 (19 + 8)		
	Meta-analytic	93.9%* [77.2–98.6%]	80.9% [51.4–94.4%]	100% [9.1–100%]		0.2201	0.1829
Decannulation, n (%)	Crude	150/291 (51.5%)	33/131 (25.2%)	117/160 (73.1%)	10 (3 + 7)		
	Meta-analytic	69.4% [39.3–88.8%]	30% [10.9–60.1%]	82.9% [53–95.4%]		0.0135	–
Duration of mechanical ventilation, days	Meta-analytic	30.9 [24.8–37.1]	26.9 [19.8–34.1]	46.1 [28.3–63.9]	22 (14 + 8)	0.0503	0.0850
			26.9 [19.8–34.1]	96.5 [65.1–127.8]^§^	22 (14 + 8)	** < 0.0001**	** < 0.0001**
ICU/rehab days	Meta-analytic	25.7 [20.2–31.2]	22.9 [17.3–28.6]	78.3 [49.1–107.6]	22 (20 + 2)	**0.0003**	**0.0003**
Hospital days	Meta-analytic	44 [37.3–50.7]	44 [37.3–50.7]	–	13 (13 + 0)	-	-
Pneumonia, n (%)	Crude	814/1927 (42.1%)	738/1630 (45.3%)	76/297 (25.6%)	21 (14 + 7)		
	Meta-analytic	38.3% [25.7–52.8%]	40.0% [27.4–54.0%]	35.5% [11.3–70.5%]		0.8132	-
Mortality, n (%)	Crude	1223/13756 (8.9%)	1210/13331 (9.1%)	13/425 (3.1%)	25 (20 + 5)		
	Meta-analytic	6.3% [4.1–9.6%]	7.7% [5.2–11.2%]	0.8% [0–18.5%]		0.1751	0.1825

Meta-analytic means and proportions were computed through the “metamean” and “metaprop” functions (R package “meta”), respectively

^*^Crude and meta-analytic proportions differed appreciably because of the very large sample size of one of the included studies, Branco et al*.* [32] (5256 patients, weighting for 73% of the total in the crude calculation), which enrolled patients with very low prevalence of tracheostomy (21%) and whose weighting in the pooled meta-analytic result was more comparable with those of the other studies

^#^*p*-values yielded by the subgroup analyses: ICUs vs rehabilitation units with and without Branco et al. [32] and Anand et al*.* [35]

^§^Including duration of mechanical ventilation prior to admission and during the stay in rehabilitation

Most studies provided no definition of weaning success/failure, and studies that reported it widely differed in their criteria; when the definition of partial weaning was provided, analogous variability was also present (Additional file 1: Table S5). The majority of the studies did not mention the use of any weaning protocol [8, 12, 31, 33, 34, 42, 47, 49, 52, 57, 61]; two studies mentioned the use of a protocol without specifying it [48, 64]. For the remaining 10 studies, the most frequently weaning protocol was a progressive increase in ventilator-free breathing, with mild variations among the studies, including unassisted breathing until tired or graded time off the ventilator [45, 50, 58, 65]; unassisted breathing alternated to periods of intermittent mandatory ventilation, continuous positive airway pressure [43] or pressure support ventilation [46, 51, 56]; non-invasive ventilation with the tracheostomy sealed [53–56]. Only 2 studies provided information on the number of patients requiring one/two or more than two weaning attempts [16, 65], and only 3 studies reported the number of patients failing extubation [31, 57, 65].

The application of rehabilitation protocols was mentioned in 5 studies and involved respiratory and physical rehabilitation [46, 56, 60], respiratory therapy [65] and respiratory muscle training [45].

### Secondary outcomes

The number of mechanical ventilation days was assessed in 14 ICU and 8 rehabilitative studies; the probability of tracheostomy in 27 studies (19 in ICU, 8 in rehabilitation), of which 10 (3 in ICU) assessed the probability of decannulation. Incidence of pneumonia was evaluated in 14 ICU and in 7 rehabilitation studies, mortality in 25 (20 ICU and 5 rehabilitation) studies (Additional file 1: Table S2).

**Fig. 3:**
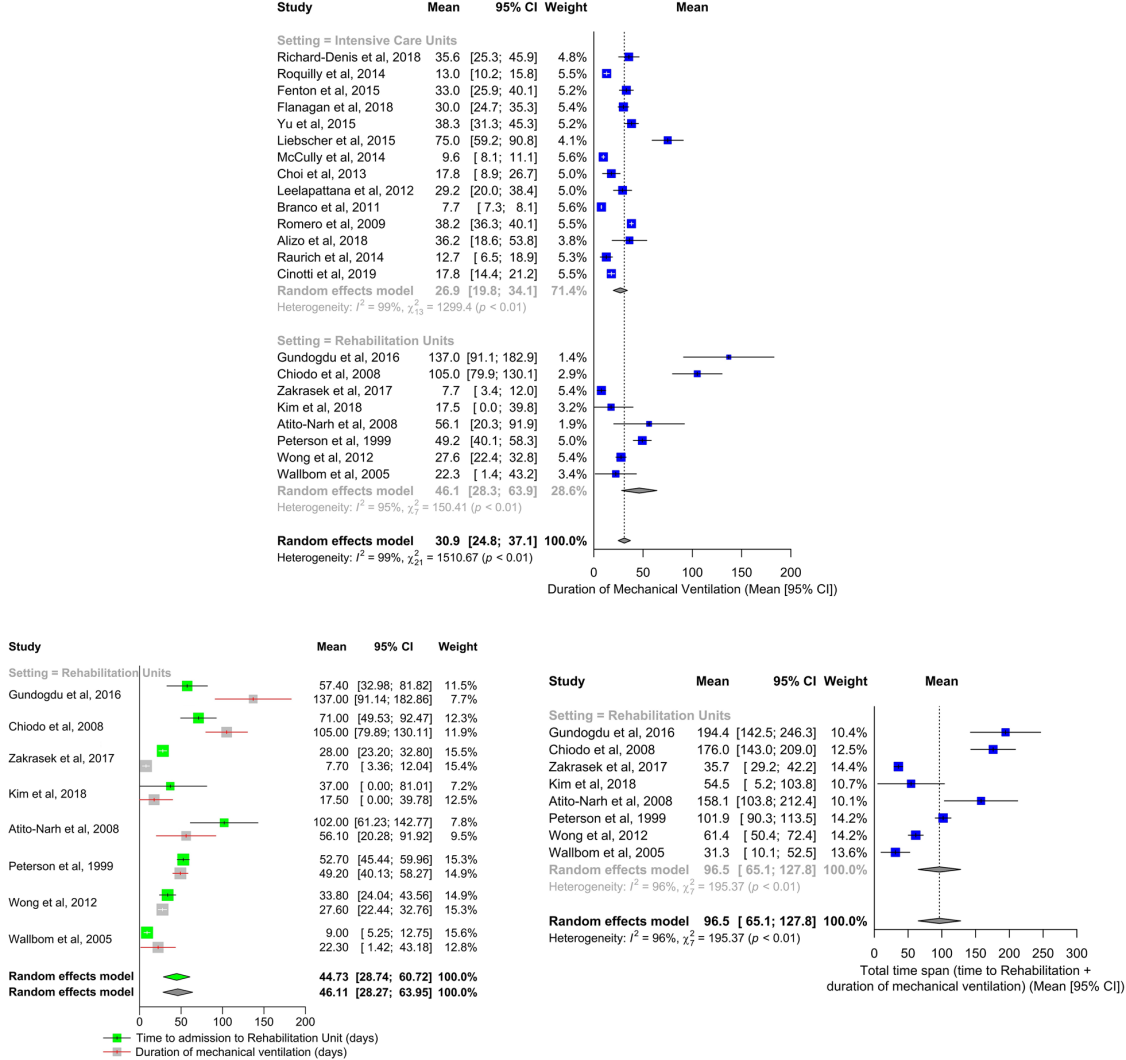
Forest plot for the outcome of duration of mechanical ventilation in intensive care units and rehabilitation units (upper panel). Studies are presented according to setting classification (intensive care units vs rehabilitation units): both overall and subgroup estimates are reported. Forest plots for the outcome of duration of mechanical ventilation for rehabilitation units (including the time to admission to rehabilitation) (lower panels). Weight refers to the relative contribution of each study to the meta-analytic estimate and is generated using the inverse variance method. CI:  confidence interval

**Fig. 4:**
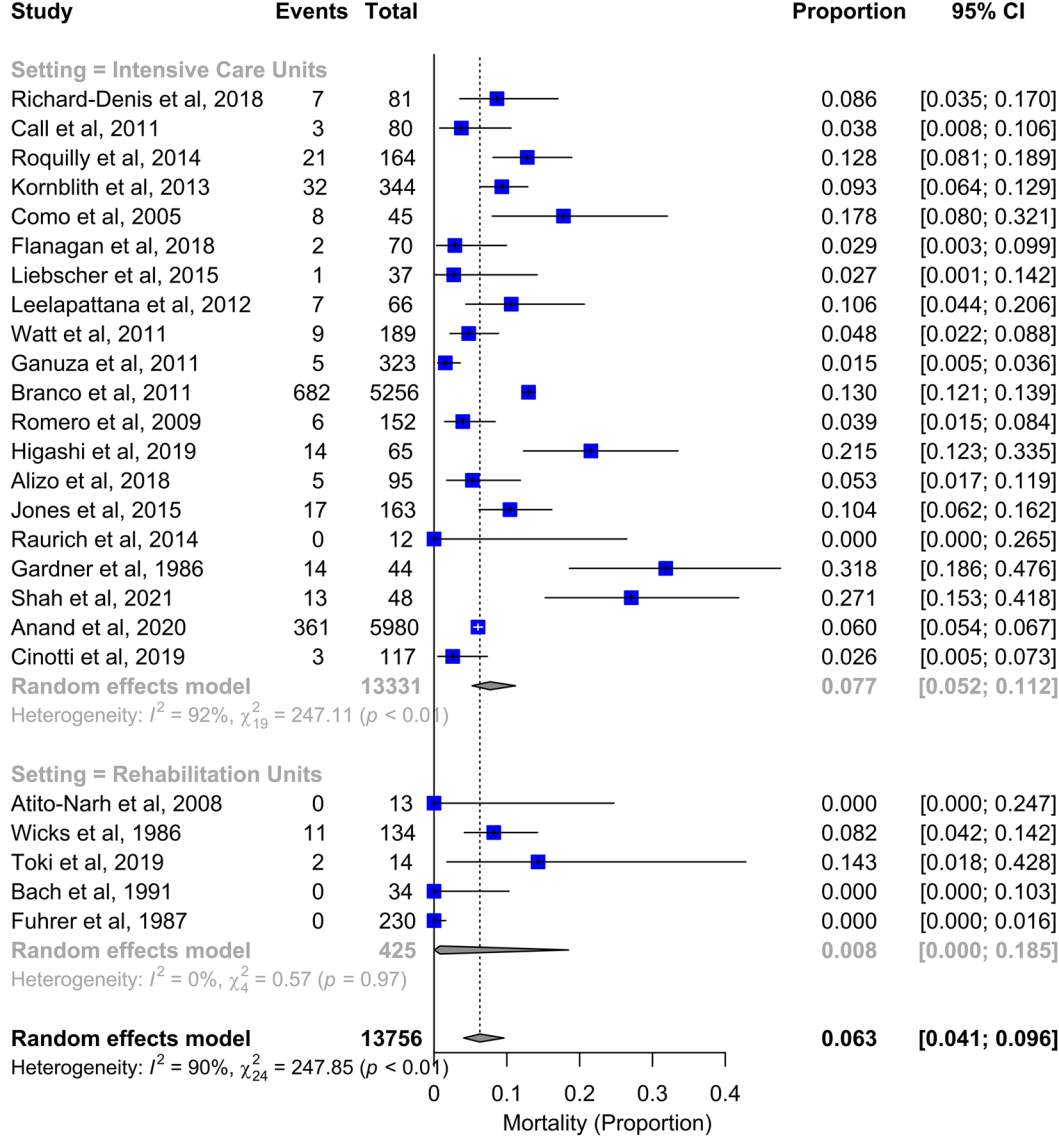
Forest plot for mortality. Studies are presented according to setting classification (intensive care units vs rehabilitation units): both overall and subgroup estimates are reported. CI: confidence interval

In ICU, the mean duration of mechanical ventilation was 27 days, length of stay 23 days, hospital stay 44 days. 81% of patients were tracheostomized and 30% of them were decannulated. Incidence of pneumonia and mortality were 40% and 8%, respectively (Figures 3 and 4, Additional file 2: Figure S1, Additional file 3: S2, Additional file 4: Figure S3, Additional file 5: Figure S4. Additional file 6: Figure S5, Table 1).

Patients hospitalized in rehabilitation centres were ventilated for a mean of 97 days (including duration of mechanical ventilation prior to admission and during the stay in rehabilitation, Fig. 3) and stayed in the unit for 78 days. All patients were tracheostomized and 83% of them were decannulated; 36% developed pneumonia, and less than 1% died (Fig. 4, Additional file 2: Figure S1, Additional file 3: S2, Additional file 3: Figure S3, Additional file 5: Figure S4. Additional file 6: Figure S5, Table 1).

### Predictors of weaning and duration of mechanical ventilation

Variables identified as predictors of weaning failure and duration of mechanical ventilation across the studies are reported in Additional file 1: Tables S6 and S7, respectively. Among all the variables considered as predictors in the different studies, we reported those that we considered clinically relevant to the outcome. A high number of comorbidities, high Injury Severity Score, high-level lesions (C1–C3 vs C4–C7), elevated heart rate, and presence of tracheostomy appeared to be associated with increased odds of weaning failure. Shorter time to admission to a specialized SCI center, high-level lesions (C1–C4 vs C5–C8), complete lesion, low tidal volume and high positive end-expiratory pressure within 24 h from admission, and presence of tracheostomy were associated to a longer duration of mechanical ventilation.

### Heterogeneity and sensitivity analysis

Data regarding heterogeneity among studies and in each subgroup are reported in Additional file 1: Table S8.

The post hoc sensitivity analysis excluding the two largest studies (Branco et al*.* [32], 5256 patients and Anand et al*.* [35], 5980 patients) did not show any difference in the duration of mechanical ventilation, proportion of tracheostomy, ICU or hospital length of stay, or mortality (Table 1, Additional file 7: Figures S6, Additional file 8: Figure S7, Additional file 9: Figure S8, Additional file 10: Figure S9, Additional file 11: Figure S10).

Due to a lack of studies reporting separate outcome data, we were unable to perform additional subgroup analyses considering the level and cause (traumatic vs non-traumatic) of spine lesion to predict the outcomes of interest. However, among the overall patients included in our study, 12,717 (87%) were retrieved by studies dealing with cervical lesions only, and of the remaining patients with mixed lesions, 1365 out of 1843 still had cervical lesions (Additional file 1: Table S4). Therefore, a potential analysis including only patients with cervical SCI was hardly expected to yield results substantially different from the overall analysis.

## Discussion

In this systematic review and meta-analysis, we found that around 63% of patients were completely weaned from mechanical ventilation in ICU after SCI, and up to 82% of the previously unweaned patients were either completely or partially liberated from the ventilator during rehabilitation. This further gain in weaning required a longer time on the ventilator and tracheostomy, but was achieved without an increased risk of pneumonia or mortality.

### Probability of weaning in ICU

The proportion of patients that were weaned in ICU varied widely among the studies, ranging from 16% [46] to more than 97% [65]. The blurred and heterogeneous definitions of weaning success across the studies make the interpretation of these findings complex. Factors likely to impact on this variability include SCI level and completeness of injury, presence of multiple spinal cord lesions, and concomitant traumatic brain injury [7]. The role of these factors is supported by the fact that some studies showing a high probability of weaning success did not include patients with SCIs higher than C3 [65], while studies reporting lower probabilities included them [8, 42, 46]. A role of these factors is also consistent with the exclusion of patients with multiple SCIs and traumatic brain injury in the studies that resulted in better weaning outcomes [65] and fewer ventilator days [30, 32, 66]. A lack of disaggregated subgroup data prevents from further investigation of these associations and identification of predictors of weaning outcomes. Univariate and multivariate regression analyses performed in some of the included studies [30, 31, 36, 37, 46, 63–65] also suggest a possible role of the severity of the lesion/trauma (reflected by the presence of a complete lesion, a high-level lesion and high Injury Severity Score), of a late transfer to a specialized acute SCI center, of clinical conditions on ICU admission (elevated heart rate, low tidal volume and high positive end-expiratory pressure values—the latter two probably reflecting a more protective strategy of ventilation), of pre-existing clinical conditions (number of comorbidities), and of the presence of tracheostomy in reducing the odds of weaning or increasing the time required to achieve it. However, studies that performed regression analyses were only a minority, the selected variables were not consistent among them, and the pooled estimates that we obtained were in most cases the result of only one or two studies. Therefore, the conclusion that can be drawn in terms of predictors of weaning outcomes are limited. Further studies are needed to better explore the association of clinical variables with weaning outcomes, and to attempt building and validating prediction models for weaning success.

### Probability of weaning in rehabilitative units

The probability of partial or complete weaning success in rehabilitation reached 82%, in some cases even attaining the totality of the patients [50, 51]. This elevated probability of successful liberation was achieved despite a greater number of patients with high-level lesions and no difference in the number of complete lesions compared to ICU studies (Additional file 1: Table S4).

There are several potential explanations for the different weaning success probabilities between acute care and rehabilitation units.

First, rehabilitation units may have selected a different population of patients transferred for late mechanical ventilation liberation. The high weaning success rate in rehabilitation units may reflect isolated high cervical SCIs, which are weaned late after resolution of acute flaccid paralysis induced by denervation, and after diaphragm training [3, 67, 68], or lower cervical and thoracic SCI lesions, which may also require prolonged mechanical ventilation, due to impaired pulmonary clearance from loss of abdominal muscle function.

Second, other concomitant injuries hindering weaning success in ICU, such as traumatic brain injury, need to recover before a patient could be transferred to rehabilitation facilities.

Third, the development of ICU-acquired weakness, potentially worsening the effect of the previous two factors, has a possible further additive role in delaying weaning until rehabilitation is started.

Finally, the application of specific rehabilitation and treatment techniques, including mechanical insufflation–exsufflation, aimed at training the respiratory muscles spared by the lesion and clearing secretions, could also favor weaning in rehabilitation units [69, 70].

### Secondary outcomes

The proportion of tracheostomy was heterogeneous, reaching unexpectedly low values in a few of the ICU studies [32, 40, 57, 66]. This is surprising considering that these studies enrolled patients with high-level lesions or older than 60 years of age [40], and because patients with SCI, due to a loss of sympathetic and expiratory muscle innervations [71], experience difficulty to clear secretions. The lack of published data and consensus regarding the optimal timing of tracheostomy in patients with SCI could explain the heterogeneity among studies. Concerns regarding the possibility of cross-contamination between tracheostomy and cervical spine fixation surgery sites have historically led to delays in tracheostomy performance [72, 73]. Not surprisingly, tracheostomized patients were found to fail weaning more and have a longer duration of mechanical ventilation. This is consistent with the finding that almost all patients admitted to rehabilitation facilities were tracheostomized.

Heterogeneity was also present in the probability of decannulation (11% to 62% in ICU and 25% to 100% in rehabilitation). This finding probably reflects the lack of published evidence and standardized criteria for decannulation in patients with SCI. Small studies and case series mainly convey the message that decannulation should be assessed on individualized basis, as these patients may never meet the traditional criteria for decannulation [74, 75].

Overall mortality was 6%, 8% when considering only patients admitted within 8 h from the injury, and less than 1% in patients admitted to rehabilitation facilities. This is low, considering life expectancy of ventilator-dependent patients after SCI [9]. One-year survival for ventilated patients with SCI was previously found to be 50%, and 25% for those admitted within 24 h from the injury [9]. Patients’ selection may explain the reduced mortality during rehabilitation since they survived the initial high-risk period in ICU [9], and rehabilitation techniques favoring weaning and reducing respiratory complications might hopefully affect mortality [69].

Incidence of pneumonia in ICU studies was in line with previous data in the acute phase of SCI [71], and was very low in rehabilitation considering the higher number of patients with C3 or above lesions and the longer duration of mechanical ventilation [69, 71].

This systematic review and meta-analysis has several strengths. It is the first systematic review to address the crucial topic of weaning in SCI and to analyze its trend separately, in ICU and during rehabilitation. It relies on a pre-registered protocol, it is based on a comprehensive literature search from inception, and it is based on a high number of patients with relatively low missing data of baseline variables. Limitations include the scarcity of randomized-controlled trials, the heterogeneity in terms of definition of weaning success/failure in the different studies and in whether they applied a weaning or rehabilitation protocol, and the lack of disaggregate data from the included studies that prevented us from performing a number of subgroup analyses.

## Conclusions

Approximately two-thirds of the patients admitted to ICU after SCI can be completely weaned off the ventilator. Weaning success can be further enhanced for patients that are admitted to rehabilitation facilities, reaching more than 80% of the previously unweaned cases, with additional days of mechanical ventilation, but no additional infectious respiratory complications or mortality. These findings support the potential usefulness of rehabilitation facilities for ventilator-dependent SCI patients, even with high cervical injuries and after a prolonged ventilation time.

This study also highlights the need to better understand the epidemiology of weaning outcomes in this category of patients, harmonize definitions, and standardize weaning procedures. This would help to elucidate the factors associated with weaning outcomes and better evaluate the role of rehabilitation. In fact, even if the application of rehabilitative techniques in specialized SCI centers, in the acute phase, is an appealing solution to improve weaning outcomes, further data are needed to support it, and to clarify the best timing, setting, and even techniques to adopt in this unique category of patients.

## Supplementary Information

**Additional file 1:  Appendix S1**. Search strategy and terms used in the electronic bibliographic databases. ** Appendix S2.** Standardized Data Collection Form used to extract data. **Table S1.** Baseline characteristics of included studies reporting study design, number of patients analyzed and included in each study, type of patients (traumatic/non-traumatic) and spine lesion level, type of ward and hospital, mean age, and mean time from injury to hospitalization. **Table S2.** Baseline characteristics of included studies II reporting inclusion and exclusion criteria of each study, assessed outcomes and presence of regression analysis. **Table S3.** The Newcastle–Ottawa Scale for assessing the quality of nonrandomized studies. **Table S4.** Baseline characteristics of the patients enrolled in each study. **Table S5.** Summary of definitions: complete weaning, partial weaning. **Table S6.** Predictors for the outcome of weaning failure. Logistic regression analyses performed across the studies are reported. Beta coefficients are pooled according to the inverse variance method; univariate and multivariate regression model coefficients are pooled together in order to obtain a robust estimate (see Complete Statistical Analysis of the Supplemental Digital Content for further details). **Table S7.** Predictors for the outcome of duration of mechanical ventilation. Linear regression analyses performed across the studies are reported. Beta coefficients are pooled according to the inverse variance method; univariate and multivariate regression model coefficients are pooled together in order to obtain a robust estimate (see Complete Statistical Analysis of the Supplemental Digital Content for further details). **Table S8.** Heterogeneity among all studies and in each subgroup (ICU and rehabilitation). Relevant heterogeneity statistics and estimate ranges are reported for characteristics at baseline, characteristics of the lesion and main outcomes after the injury.

**Additional file 2:Figure S1.** Forest plot for the probability of tracheostomy. Studies are presented according to setting classification (Intensive Care Units vs Rehabilitation Units): both overall and subgroup estimates are reported. CI = confidence interval.

**Additional file 3: Figure S2.** Forest plot for the probability of decannulation after tracheostomy. Studies are presented according to setting classification (Intensive Care Units vs Rehabilitation Units): both overall and subgroup estimates are reported. CI = confidence interval.

**Additional file 4: Figure S3.** Forest plot for Intensive Care Unit or Rehabilitation Unit lengths of stay. Studies are presented according to setting classification: both overall and subgroup estimates are reported. Weight refers to the relative contribution of each study to the meta-analytic estimate and is generated using the inverse variance method. ICU = Intensive Care Unit, CI = confidence interval.

**Additional file 5: Figure S4.** Forest plot for hospital length of stay in Intensive Care Unit setting (no data are available for Rehabilitation Units). Weight refers to the relative contribution of each study to the meta-analytic estimate and is generated using the inverse variance method. CI = confidence interval.

**Additional file 6: Figure S5.** Forest plot for the probability of pneumonia. Studies are presented according to setting classification (Intensive Care Units vs Rehabilitation Units): both overall and subgroup estimates are reported. CI = confidence interval.

**Additional file 7: Figure S6.** Sensitivity analysis: Forest plot for the outcome of duration of MV (complete liberation from the ventilator) excluding the two largest studies (Branco et al*.* [32] and Anand et al*.* [35]). CI = confidence interval.

**Additional file 8: Figure S7.** Sensitivity analysis: Forest plot for the probability of tracheostomy excluding the two largest studies (Branco et al*.* [32] and Anand et al. [35]). CI = confidence interval.

**Additional file 9: Figure S8.** Sensitivity analysis: Forest plot for Intensive Care Unit or Rehabilitation Unit lengths of stay excluding the two largest studies (Branco et al. [32] and Anand et al. [35]). ICU = Intensive Care Unit, CI = confidence interval.

**Additional file 10: Figure S9.** Sensitivity analysis: Forest plot for hospital length of stay excluding the two largest studies (Branco et al. [32] and Anand et al. [35]). CI = confidence interval.

**Additional file 11: Figure S10.** Sensitivity analysis: Forest plot for mortality excluding the two largest studies (Branco et al. [32] and Anand et al. [35]). CI = confidence interval.

## Data Availability

The datasets analyzed during the current study are available from the corresponding author on reasonable request.

## Abbreviations

CI: Confidence interval
ICU: Intensive care unit
Q1–Q3: 1st–3rd quartile intervals
SCI: Spinal cord injury
SD: Standard deviation
