# Supplementary material for: Separation from mechanical ventilation and survival after spinal cord injury: a systematic review and meta-analysis
Source: Ann Intensive Care. 2021 Oct 24;11:149. doi: 10.1186/s13613-021-00938-x (PMC8542415; doi:10.1186/s13613-021-00938-x)
Supplement: Supplementary file 1 — Additional file 1: Appendix S1. Search strategy and terms used in the electronic bibliographic databases. Appendix S2. Standardized Data Collection Form used to extract data. Table S1. Baseline characteristics of included studies reporting study design, number of patients analyzed and included in each study, type of patients (traumatic/non-traumatic) and spine lesion level, type of ward and hospital, mean age, and mean time from injury to hospitalization. Table S2. Baseline characteristics of included studies II reporting inclusion and exclusion criteria of each study, assessed outcomes and presence of regression analysis. Table S3. The Newcastle–Ottawa Scale for assessing the quality of nonrandomized studies. Table S4. Baseline characteristics of the patients enrolled in each study. Table S5. Summary of definitions: complete weaning, partial weaning. Table S6. Predictors for the outcome of weaning failure. Logistic regression analyses performed across the studies are reported. Beta coefficients are pooled according to the inverse variance method; univariate and multivariate regression model coefficients are pooled together in order to obtain a robust estimate (see Complete Statistical Analysis of the Supplemental Digital Content for further details). Table S7. Predictors for the outcome of duration of mechanical ventilation. Linear regression analyses performed across the studies are reported. Beta coefficients are pooled according to the inverse variance method; univariate and multivariate regression model coefficients are pooled together in order to obtain a robust estimate (see Complete Statistical Analysis of the Supplemental Digital Content for further details). Table S8. Heterogeneity among all studies and in each subgroup (ICU and rehabilitation). Relevant heterogeneity statistics and estimate ranges are reported for characteristics at baseline, characteristics of the lesion and main outcomes after the injury. [file 13613_2021_938_MOESM1_ESM.docx]

#

# SUPPLEMENTAL DIGITAL CONTENT

**Separation from mechanical ventilation and outcomes in patients with spinal cord injury: a systematic review and meta-analysis**

Annia F. Schreiber, MD, Jacopo Garlasco, MD, Fernando Vieira, PT, MSc, Yie Hui Lau, MD, Dekel Stavi, MD, David Lightfoot, PhD, Andrea Rigamonti, MD, Karen Burns, MD, FRCPC, MSc, Jan O. Friedrich, MD, DPhil, MSc, Jeffrey M. Singh, MD, MSc, FRCPC, Laurent J. Brochard, MD, HDR

**Supplemental Appendix 1.** Search Strategy and terms used in the electronic bibliographic databases

**Supplemental Appendix 2.** Standardized Data Collection Form used to extract data

**Complete Statistical analysis**

**e-Table 1.** Baseline characteristics of included studies reporting study design, number of patients analyzed and included in each study, type of patients (traumatic/non-traumatic) and spine lesion level, type of ward and hospital, mean age, and mean time from injury to hospitalization

**e-Table 2.** Baseline characteristics of included studies II, reporting: inclusion and exclusion criteria of each study, assessed outcomes and presence of regression analysis

**e-Table 3.** The Newcastle-Ottawa Scale for assessing the quality of nonrandomized studies

**e-Table 4.** Baseline characteristics of the patients enrolled in each study

**e-Table 5.** Summary of definitions: complete weaning, partial weaning

**e-Table 6.** Predictors for the outcome of weaning failure. Logistic regression analyses performed across the studies are reported. Beta coefficients are pooled according to the inverse variance method; univariate and multivariate regression model coefficients are pooled together in order to obtain a robust estimate (see Complete Statistical Analysis of the Supplemental Digital Content for further details)

**e-Table 7.** Predictors for the outcome of duration of mechanical ventilation. Linear regression analyses performed across the studies are reported. Beta coefficients are pooled according to the inverse variance method; univariate and multivariate regression model coefficients are pooled together in order to obtain a robust estimate (see Complete Statistical Analysis of the Supplemental Digital Content for further details)

**e-Table 8.** Heterogeneity among all studies and in each subgroup (ICU and rehabilitation). Relevant heterogeneity statistics and estimate ranges are reported for characteristics at baseline, characteristics of the lesion and main outcomes after the injury

**References**

**Supplemental Appendix 1. Search Strategy**

The following tables record the search strategy and terms used in each of the electronic bibliographic databases. The search was conducted to identify randomized and quasi-randomized controlled trials and observational studies from the inception of each of the databases to August 2021. No language restriction, nor limit to gender or race were applied.

##

## *OVID Medline*

Database: All Ovid Medline <1946 – August 2021>

Search Strategy:

--------------------------------------------------------------------------------

exp Spinal Cord Injuries/ (49668)

2 (spinal cord adj5 Contusion*).ti,ab,kf. (1176)

3 (spine adj5 Contusion*).ti,ab,kf. (32)

4 (spinal adj5 injur*).ti,ab,kf. (49908)

5 (spine adj5 injur*).ti,ab,kf. (7310)

6 (spinal adj5 trauma*).ti,ab,kf. (8369)

7 (spine adj5 trauma*).ti,ab,kf. (3358)

8 (spinal adj5 lacerat*).ti,ab,kf. (60)

9 (spine adj5 lacerat*).ti,ab,kf. (20)

10 (myelopath* adj5 traumatic).ti,ab,kf. (170)

11 (myelopath* adj5 postraumatic).ti,ab,kf. (1)

12 exp Paraplegia/ (13109)

13 paraplegi*.ti,ab,kf. (17623)

14 parapleg*.ti,ab,kf. (17625)

15 (paraparesis or parapareses).ti,ab,kf. (6136)

16 exp Quadriplegia/ (8116)

17 quadriplegi*.ti,ab,kf. (4294)

18 (quadriparesis or quadripareses).ti,ab. (1516)

19 tetraplegi*.ti,ab,kf. (4448)

20 (tetraparesis or tetrapareses).ti,ab. (1388)

21 or/1-20 (105392)

22 exp ventilators, mechanical/ (9351)

23 (Elisee or PLV 100 or Respironics or Servo-air or Tangens or Trilogy).tw. (823)

24 exp Respiration, Artificial/ (79571)

25 exp Respiratory Insufficiency/ (63715)

26 (respirat$ adj2 invasive).ti,ab,kf. (543)

27 (respirat$ adj2 mechanical).ti,ab,kf. (853)

28 (respirat$ adj2 automated$).ti,ab,kf. (58)

29 (respirat* adj2 negative pressure).ti,ab,kf. (79)

30 (respirat* adj2 jet).ti,ab,kf. (13)

31 (ventilat$ adj2 patient).mp. [mp=title, abstract, original title, name of substance word, subject heading word, floating sub-heading word, keyword heading word, organism supplementary concept word, protocol supplementary concept word, rare disease supplementary concept word, unique identifier, synonyms] (2449)

32 exp ventilator weaning/ (4009)

33 (ventilat$ adj2 wean$).ti,ab,kf. (1464)

34 (ventilat$ adj wean$).m_titl. (226)

35 (ventilat$ adj2 discontin$).ti,ab,kf. (261)

36 (ventilat$ adj2 extubat*$).ti,ab,kf. (333)

37 (ventilat*$ adj2 invasive).ti,ab,kf. (6800)

38 respiratory fail*.ti,ab,kf. (33553)

39 (respiratory adj5 fail*).ti,ab,kf. (37213)

40 (respiratory adj5 insufficien*).ti,ab,kf. (9788)

41 Breath Tests/ (15166)

42 breathing trial*.ti,ab,kf. (733)

43 extubation, Intratracheal/ (1692)

44 device removal/mt (3565)

45 Respiratory Muscles/ (5527)

46 exp Breath Tests/ (15166)

47 (breath analysis or breath analyses).ti,ab,kf. (1204)

48 (breath* adj2 test*).ti,ab,kf. (9444)

49 breathing trial.mp. (482)

50 (breath* adj2 trial*).ti,ab,kf. (868)

51 device removal/ (13767)

52 devise remov*.ti,ab,kf. (1)

53 or/22-52 (206576)

54 21 and 53 (2113)

55 limit 54 to ("newborn infant (birth to 1 month)" or "infant (1 to 23 months)" or "preschool child (2 to 5 years)" or "child (6 to 12 years)") (309)

56 child/ (1721126)

57 55 or 56 (1721214)

58 54 not 57 (1804)

59 limit 54 to ("young adult (19 to 24 years)" or "adult (19 to 44 years)" or "young adult and adult (19-24 and 19-44)" or "middle age (45 to 64 years)" or "middle aged (45 plus years)" or "all aged (65 and over)" or "aged (80 and over)") (1252)

60 58 or 59 (1931)

61 animals/ not (humans/ and animals/) (4757468)

62 60 not 61 (1835)

63 limit 62 to (address or case reports or comment or duplicate publication or festschrift or lecture or legal case or legislation or letter) (683)

64 62 not 63 (1152)

65 remove duplicates from 64 (1152)

66 limit 65 to ed=20191001-20211212 (72)

67 65 not 66 (1177)

## *CINAHL*

| S19 | S17 NOT S18 |  | [**View Results**](javascript:__doPostBack('ctl00$ctl00$FindField$FindField$historyControl$HistoryRepeater$ctl00$linkResults','')) (584)  [**View Details**](javascript:showShDetails(%22ctl00_ctl00_FindField_FindField_historyControl_ctrlPopup%22,%20%22S19%22);)  [**Edit**](http://web.b.ebscohost.com/Legacy/Views/UserControls/Ehost/) | | |
| --- | --- | --- | --- | --- | --- |
| S18 | (MM "Case Studies") OR "case report" |  | [**View Results**](javascript:__doPostBack('ctl00$ctl00$FindField$FindField$historyControl$HistoryRepeater$ctl01$linkResults','')) (56,677)  [**View Details**](javascript:showShDetails(%22ctl00_ctl00_FindField_FindField_historyControl_ctrlPopup%22,%20%22S18%22);)  [**Edit**](http://web.b.ebscohost.com/Legacy/Views/UserControls/Ehost/) | | |
| S17 | S7 AND S15 |  | [**View Results**](javascript:__doPostBack('ctl00$ctl00$FindField$FindField$historyControl$HistoryRepeater$ctl02$linkResults','')) (602)  [**View Details**](javascript:showShDetails(%22ctl00_ctl00_FindField_FindField_historyControl_ctrlPopup%22,%20%22S17%22);)  [**Edit**](http://web.b.ebscohost.com/Legacy/Views/UserControls/Ehost/) | | |
| S16 | S7 AND S15 |  | [**View Results**](javascript:__doPostBack('ctl00$ctl00$FindField$FindField$historyControl$HistoryRepeater$ctl03$linkResults','')) (1,913)  [**View Details**](javascript:showShDetails(%22ctl00_ctl00_FindField_FindField_historyControl_ctrlPopup%22,%20%22S16%22);)  [**Edit**](http://web.b.ebscohost.com/Legacy/Views/UserControls/Ehost/) | | |
| S15 | S8 OR S9 OR S10 OR S11 OR S12 OR S13 OR S14 |  | [**View Results**](javascript:__doPostBack('ctl00$ctl00$FindField$FindField$historyControl$HistoryRepeater$ctl04$linkResults','')) (56,107)  [**View Details**](javascript:showShDetails(%22ctl00_ctl00_FindField_FindField_historyControl_ctrlPopup%22,%20%22S15%22);)  [**Edit**](http://web.b.ebscohost.com/Legacy/Views/UserControls/Ehost/) | | |
| S14 | (MH "Device Removal+") |  | [**View Results**](javascript:__doPostBack('ctl00$ctl00$FindField$FindField$historyControl$HistoryRepeater$ctl05$linkResults','')) (4,951)  [**View Details**](javascript:showShDetails(%22ctl00_ctl00_FindField_FindField_historyControl_ctrlPopup%22,%20%22S14%22);)  [**Edit**](http://web.b.ebscohost.com/Legacy/Views/UserControls/Ehost/) | | |
| S13 | (MM "Extubation") OR "extubation" |  | [**View Results**](javascript:__doPostBack('ctl00$ctl00$FindField$FindField$historyControl$HistoryRepeater$ctl06$linkResults','')) (3,443)  [**View Details**](javascript:showShDetails(%22ctl00_ctl00_FindField_FindField_historyControl_ctrlPopup%22,%20%22S13%22);)  [**Edit**](http://web.b.ebscohost.com/Legacy/Views/UserControls/Ehost/) | | |
| S12 | (MM "Breath Tests") OR "breathing test" |  | [**View Results**](javascript:__doPostBack('ctl00$ctl00$FindField$FindField$historyControl$HistoryRepeater$ctl07$linkResults','')) (762)  [**View Details**](javascript:showShDetails(%22ctl00_ctl00_FindField_FindField_historyControl_ctrlPopup%22,%20%22S12%22);)  [**Edit**](http://web.b.ebscohost.com/Legacy/Views/UserControls/Ehost/) | | |
| S11 | TX ventilator OR respirator or "ventilated patient" |  | [**View Results**](javascript:__doPostBack('ctl00$ctl00$FindField$FindField$historyControl$HistoryRepeater$ctl08$linkResults','')) (29,843)  [**View Details**](javascript:showShDetails(%22ctl00_ctl00_FindField_FindField_historyControl_ctrlPopup%22,%20%22S11%22);)  [**Edit**](http://web.b.ebscohost.com/Legacy/Views/UserControls/Ehost/) | | |
| S10 | (MH "Respiratory Failure+") |  | [**View Results**](javascript:__doPostBack('ctl00$ctl00$FindField$FindField$historyControl$HistoryRepeater$ctl09$linkResults','')) (13,027)  [**View Details**](javascript:showShDetails(%22ctl00_ctl00_FindField_FindField_historyControl_ctrlPopup%22,%20%22S10%22);)  [**Edit**](http://web.b.ebscohost.com/Legacy/Views/UserControls/Ehost/) | | |
| S9 | TX Elisee or PLV 100 or Respironics or Servo-air or Tangens or Trilogy) |  | [**View Results**](javascript:__doPostBack('ctl00$ctl00$FindField$FindField$historyControl$HistoryRepeater$ctl10$linkResults','')) (2,167)  [**View Details**](javascript:showShDetails(%22ctl00_ctl00_FindField_FindField_historyControl_ctrlPopup%22,%20%22S9%22);)  [**Edit**](http://web.b.ebscohost.com/Legacy/Views/UserControls/Ehost/) | | |
| S8 | (MM "Ventilators, Mechanical") OR (MM "Ventilator Weaning") OR (MM "Respiration, Artificial") |  | [**View Results**](javascript:__doPostBack('ctl00$ctl00$FindField$FindField$historyControl$HistoryRepeater$ctl11$linkResults','')) (12,880)  [**View Details**](javascript:showShDetails(%22ctl00_ctl00_FindField_FindField_historyControl_ctrlPopup%22,%20%22S8%22);)  [**Edit**](http://web.b.ebscohost.com/Legacy/Views/UserControls/Ehost/) | |  |
| S7 | S1 OR S2 OR S3 OR S4 OR S5 OR S6 |  | [**View Results**](javascript:__doPostBack('ctl00$ctl00$FindField$FindField$historyControl$HistoryRepeater$ctl12$linkResults','')) (65,375)  [**View Details**](javascript:showShDetails(%22ctl00_ctl00_FindField_FindField_historyControl_ctrlPopup%22,%20%22S7%22);)  [**Edit**](http://web.b.ebscohost.com/Legacy/Views/UserControls/Ehost/) | |  |
| S6 | TX tetraplagi* or tetraparesis or tetrapareses |  | [**View Results**](javascript:__doPostBack('ctl00$ctl00$FindField$FindField$historyControl$HistoryRepeater$ctl13$linkResults','')) (274)  [**View Details**](javascript:showShDetails(%22ctl00_ctl00_FindField_FindField_historyControl_ctrlPopup%22,%20%22S6%22);)  [**Edit**](http://web.b.ebscohost.com/Legacy/Views/UserControls/Ehost/) | |  |
| S5 | TX quadriparesis or quadripareses |  | [**View Results**](javascript:__doPostBack('ctl00$ctl00$FindField$FindField$historyControl$HistoryRepeater$ctl14$linkResults','')) (499)  [**View Details**](javascript:showShDetails(%22ctl00_ctl00_FindField_FindField_historyControl_ctrlPopup%22,%20%22S5%22);)  [**Edit**](http://web.b.ebscohost.com/Legacy/Views/UserControls/Ehost/) | |  |
| S4 | TX paraplegia OR paraparesis OR parapareses |  | [**View Results**](javascript:__doPostBack('ctl00$ctl00$FindField$FindField$historyControl$HistoryRepeater$ctl15$linkResults','')) (7,039)  [**View Details**](javascript:showShDetails(%22ctl00_ctl00_FindField_FindField_historyControl_ctrlPopup%22,%20%22S4%22);)  [**Edit**](http://web.b.ebscohost.com/Legacy/Views/UserControls/Ehost/) | |  |
| S3 | (MH "Paraplegia+") |  | [**View Results**](javascript:__doPostBack('ctl00$ctl00$FindField$FindField$historyControl$HistoryRepeater$ctl16$linkResults','')) (2,727)  [**View Details**](javascript:showShDetails(%22ctl00_ctl00_FindField_FindField_historyControl_ctrlPopup%22,%20%22S3%22);)  [**Edit**](http://web.b.ebscohost.com/Legacy/Views/UserControls/Ehost/) | |  |
| S2 | TX "spinal cord Contusion" OR "spine contusion" OR "spinal injur*" OR "spine injur*" OR "spinal cord" OR "spinal laceration" OR "spine laceration" OR "myelopathy" OR "myelopathies" |  | [**View Results**](javascript:__doPostBack('ctl00$ctl00$FindField$FindField$historyControl$HistoryRepeater$ctl17$linkResults','')) (56,562)  [**View Details**](javascript:showShDetails(%22ctl00_ctl00_FindField_FindField_historyControl_ctrlPopup%22,%20%22S2%22);)  [**Edit**](http://web.b.ebscohost.com/Legacy/Views/UserControls/Ehost/) | |  |
| S1 | (MH "Spinal Cord Injuries+") OR "Spinal Cord Injuries" OR (MH "Spinal Injuries+") | [**View Results**](javascript:__doPostBack('ctl00$ctl00$FindField$FindField$historyControl$HistoryRepeater$ctl18$linkResults','')) (27,864) | |  |  |

## *EBM Reviews - Cochrane Central Register of Controlled Trials, EBM Reviews - Cochrane Database of Systematic Reviews*

Database: EBM Reviews - Cochrane Central Register of Controlled Trials <August 2021>, EBM Reviews - Cochrane Database of Systematic Reviews <2005 to August 2021>

Search Strategy:

--------------------------------------------------------------------------------

1 exp Spinal Cord Injuries/ (1495)

2 [(spinal cord adj5 Contusion*).ti,ab,kf.] (0)

3 [(spine adj5 Contusion*).ti,ab,kf.] (0)

4 [(spinal adj5 injur*).ti,ab,kf.] (0)

5 [(spine adj5 injur*).ti,ab,kf.] (0)

6 [(spinal adj5 trauma*).ti,ab,kf.] (0)

7 [(spine adj5 trauma*).ti,ab,kf.] (0)

8 [(spinal adj5 lacerat*).ti,ab,kf.] (0)

9 [(spine adj5 lacerat*).ti,ab,kf.] (0)

10 [(myelopath* adj5 traumatic).ti,ab,kf.] (0)

11 [(myelopath* adj5 postraumatic).ti,ab,kf.] (0)

12 exp Paraplegia/ (199)

13 [paraplegi*.ti,ab,kf.] (0)

14 [parapleg*.ti,ab,kf.] (0)

15 [(paraparesis or parapareses).ti,ab,kf.] (0)

16 exp Quadriplegia/ (181)

17 [quadriplegi*.ti,ab,kf.] (0)

18 (quadriparesis or quadripareses).ti,ab. (10)

19 [tetraplegi*.ti,ab,kf.] (0)

20 (tetraparesis or tetrapareses).ti,ab. (13)

21 or/1-20 (1683)

22 exp Pulmonary Ventilation/ (6984)

23 exp ventilators, mechanical/ (257)

24 (Elisee or PLV 100 or Respironics or Servo-air or Tangens or Trilogy).tw. (279)

25 exp Respiration, Artificial/ (5720)

26 exp Respiratory Insufficiency/ (2379)

27 [(respirat$ adj2 invasive).ti,ab,kf.] (0)

28 [(respirat$ adj2 mechanical).ti,ab,kf.] (0)

29 [(respirat$ adj2 automated$).ti,ab,kf.] (0)

30 [(respirat* adj2 negative pressure).ti,ab,kf.] (0)

31 [(respirat* adj2 jet).ti,ab,kf.] (0)

32 (ventilat$ adj2 patient).mp. [mp=ti, ot, ab, sh, hw, kw, tx, ct] (1370)

33 exp ventilator weaning/ (464)

34 [(ventilat$ adj2 wean$).ti,ab,kf.] (0)

35 (ventilat$ adj wean$).m_titl. (103)

36 [(ventilat$ adj2 discontin$).ti,ab,kf.] (0)

37 [(ventilat$ adj2 extubat*$).ti,ab,kf.] (0)

38 [(ventilat*$ adj2 invasive).ti,ab,kf.] (0)

39 [respiratory fail*.ti,ab,kf.] (0)

40 [(respiratory adj5 fail*).ti,ab,kf.] (0)

41 [(respiratory adj5 insufficien*).ti,ab,kf.] (0)

42 Breath Tests/ (1514)

43 [breathing trial*.ti,ab,kf.] (0)

44 extubation, Intratracheal/ (198)

45 device removal/mt (0)

46 Respiratory Muscles/ (527)

47 exp Breath Tests/ (1514)

48 [(breath analysis or breath analyses).ti,ab,kf.] (0)

49 [(breath* adj2 test*).ti,ab,kf.] (0)

50 breathing trial.mp. (222)

51 [(breath* adj2 trial*).ti,ab,kf.] (0)

52 device removal/ (380)

53 [devise remov*.ti,ab,kf.] (0)

54 or/22-53 (17796)

55 21 and 54 (41)

56 animals/ not (humans/ and animals/) (3)

57 55 not 56 (41)

# *Embase Classic+Embase <1947 to August 30, 2021>*

Search Strategy:

--------------------------------------------------------------------------------

1 exp spinal cord injury/ (82960)

2 Spinal cord injur*.ti,ab. [truncated to search for injury or injuries in the title and abstract] (52774)

3 (spinal adj5 Contusion*).ti,ab. (1705)

4 (spine adj5 Contusion*).ti,ab. (58)

5 (spinal adj5 injur*).ti,ab. (63603)

6 (spine adj5 injur*).ti,ab. (8747)

7 (spinal adj5 trauma*).ti,ab. (11180)

8 (spine adj5 trauma*).ti,ab. (4298)

9 (spinal adj5 lacerat*).ti,ab. (76)

10 (spine adj5 lacerat*).ti,ab. (24)

11 (myelopath* adj5 traumatic).ti,ab. (227)

12 (myelopath* adj5 posttraumatic).ti,ab. (51)

13 (myelopath* adj5 post traumatic).ti,ab. (46)

14 paraplegia/ (28557)

15 paraplegi*.ti,ab. (24776)

16 (paraparesis or parapareses).ti,ab. (8489)

17 Quadriplegia/ (19398)

18 Quadriplegi*.ti,ab. (6321)

19 (quadriparesis or quadripareses).ti,ab. (2241)

20 tetraplegi*.ti,ab. (6367)

21 (tetraparesis or tetrapareses).ti,ab. (2268)

22 or/1-21 (153452)

23 exp artificial ventilation/ (226583)

24 (artificial adj2 ventilat*).ti,ab. (5414)

25 exp mechanical ventilator/ (4153)

26 ventilator.ti,ab. (42154)

27 Elisee.ti,ab. (23)

28 PLV-100.ti,ab. (8)

29 Respironics.ti,ab. (872)

30 Servo-air.ti,ab. (0)

31 Tangens.ti,ab. (5)

32 Trilogy.ti,ab. (1067)

33 (respirat$ adj2 invasive).ti,ab. (974)

34 (respirat$ adj2 mechanical).ti,ab. (1475)

35 (respirat$ adj2 automated$).ti,ab. (78)

36 (respirat* adj2 negative pressure).ti,ab. (134)

37 (respirat* adj2 jet).ti,ab. (18)

38 (respirat* adj2 jet).ti,ab. (18)

39 exp respiratory failure/ (114285)

40 Respiratory Insufficienc*.ti,ab. (12654)

41 Respiratory fail*.ti,ab. (57431)

42 exp breathing muscle/ (13377)

43 breathing Muscle*.ti,ab. (128)

44 Respiratory Muscle*.ti,ab. (10884)

45 exp breath analysis/ (20889)

46 breath test*.ti,ab. (12150)

47 (breath* adj2 test*).ti,ab. (14861)

48 (breath* adj2 trial*).ti,ab. (1598)

49 devise remov*.ti,ab. (1)

50 device removal/ (20857)

51 exp ventilator weaning/ (2273)

52 ventilator wean*.ti,ab. (778)

53 (ventilat$ adj2 discontin$).ti,ab. (387)

54 (ventilat$ adj2 extubat*$).ti,ab. (572)

55 or/23-54 (400341)

56 22 and 55 (5709)

57 limit 56 to (infant <to one year> or child <unspecified age> or preschool child <1 to 6 years> or school child <7 to 12 years> or adolescent <13 to 17 years>) (954)

58 56 not 57 (4755)

59 limit 56 to (adult <18 to 64 years> or aged <65+ years>) (3088)

60 58 or 59 (5143)

61 limit 60 to human (4597)

62 limit 61 to (books or chapter or conference abstract or conference paper or "conference review" or editorial or erratum or letter or note) (1249)

63 case report.mp. or exp case report/ (2741948)

64 62 or 63 (2742751)

65 61 not 64 (2071)

66 limit 65 to dd=20191001-20211212 (31)

67 65 not 66 (2094)

## *Scopus 5547*

( TITLE-ABS-KEY ( spinal  AND cord  AND injur*  OR  spine  AND injuries  OR  spinal  AND trauma  OR  spine  AND trauma  OR  myelopath*  OR  paraplag*  OR  quadraplag* )  AND  TITLE-ABS-KEY ( ventilation  OR  respiration  OR  ventilator*  OR  respirator  OR  breathing  OR  breath  OR  extubat* ) )

**Supplemental Appendix 2 – Data Abstraction Form**

***Data Abstraction Form – SCI weaning outcomes SR***

Name of data abstractor (first, last): _________________ ___________________

**1. Study ID**

Study Identification Number _________________________ Date ______________________

Study Title _______________________________________________________________________

First Author Name, Year of Publication _______________________________ _____________

Journal Name ____________________________________________________________________

Contact information (email address of first author preferred) _________________________________

**Is this an abstract publication (never published as a full manuscript)?** ❑ No ❑ Yes

**2. Study Eligibility**

**a. Study design**

Is the study a randomized controlled trial? ❑Yes ❑No

If yes,

- Is the study clearly randomized? ❑Yes ❑Unclear ❑No
- Is the study pseudo-randomized? ❑Yes ❑Unclear ❑No

Is the study an observational study? ❑Yes ❑No

If yes,

- Is the study retrospective? ❑Yes ❑No

Is it a physiological study? ❑Yes ❑Unclear ❑No

If yes,

- Does it include a clinical outcome? ❑Yes ❑Unclear ❑No

**b. Study Participants**

Do the participants have SCI? ❑Yes ❑Unclear ❑No

Are at least 80% of the participants

invasively ventilated? ❑Yes ❑Unclear ❑No

If yes,

- Are the participants ventilator-dependent? ❑Yes ❑Unclear ❑No

Are the participants non-invasively ventilated? ❑Yes ❑Unclear ❑No

Are at least 80% of the participants adults ❑Yes ❑Unclear ❑No

(> 16 years of age)?

**c. Study Intervention**

Did the study include patients treated with invasive

mechanical ventilation? ❑Yes ❑Unclear ❑No

Was the possibility of weaning assessed? ❑Yes ❑Unclear ❑No

**d. Study Outcomes**

Did the study report any of the following outcomes?

- Number of patients successfully weaned ❑Yes ❑Unclear ❑No

If yes, do we have information on:

- N° of patients completely liberated ❑Yes ❑Unclear ❑No

from the ventilator?

- N° of patients completely dependent from ❑Yes ❑Unclear ❑No

the ventilator?

- N° of patients partially dependent from ❑Yes ❑Unclear ❑No

the ventilator?

- Time to partial or complete liberation ❑Yes ❑Unclear ❑No

from the ventilator

- Factors associated with the other two ❑Yes ❑Unclear ❑No

outcomes (number of patients weaned

and time to liberation)

- Length of stay (hospital/ICU) ❑Yes ❑Unclear ❑No

Are the participants similar in all important

respects except for the exposure? ❑Yes ❑Unclear ❑No

Was the allocation sequence adequately ❑Adequate ❑Unclear ❑Inadequate

generated?

**Method of randomization**  Specify_______________________________________

Describe the method used or randomization

(Circle grade) ❑Adequate ❑Unclear ❑Inadequate

**Time of randomization** Specify________________________________________

(e.g., admission, upon meeting criteria) ______________________________________________

**Allocation concealment** Specify_______________________________________

Describe the method used to conceal _____________________________________________

random allocation sequence. (Circle grade)

❑Adequate ❑Unclear ❑Inadequate ❑Not used

- Mortality at any time ❑Yes ❑Unclear ❑No

**e. Inclusion Criteria**

Did the study include at least 80% adults with SCI ❑Yes ❑Unclear ❑No

(at any level and from any cause) requiring

invasive mechanical ventilation?

**f. Exclusion Criteria**

Did the study include more than 20% of

adolescents or children under 16 years of age? ❑Yes ❑Unclear ❑No

Is the study a case report/case series < 10 patients ❑Yes ❑Unclear ❑No

**3. Information source**

How was the article/abstract identified?

**Does the study meet all of the above criteria and meet none of the exclusion criteria? ❑Yes ❑No**

**Decision:** **❑Include (Go to point 3) ❑Exclude, reason _____________________________________**

**❑Additional information is required before a decision can be made**

**Possible article for inclusion in Pacing Systematic Review? ❑Yes ❑No**

**3. Potential Sources of Bias**

**Adequate/Yes (criteria appropriately applied and described in the report or acknowledged from the primary author of the study);
Unclear (criteria not described or impossible to acquire from the author);
Inadequate/No (criteria inappropriately applied)**

1. **For randomized controlled trials**

- **Selection bias**
- **Information/Detection bias**

Was information about outcome obtained ❑Yes ❑Unclear ❑No

in the same way for all the participants?

**Outcome assessor blinding?** Specify_______________________________________

Were outcomes assessors independent ______________________________________________

from individuals administering or supervising _____________________________________________

the assigned interventions? (Circle grade) ❑Yes ❑Unclear ❑No

- **Attrition/Transfer bias**

**Dropouts/withdrawals?**

Were any withdrawals/dropouts described? ❑Yes ❑Unclear ❑No

Did they occur with similar frequency

between study groups? (Circle grade) ❑Yes ❑Unclear ❑No

**Intention to treat analysis?**

Were all patients analyzed according to the group ❑All participants entered into trial

they were initially assigned to whether they received ❑15% or fewer excluded

it or not? (Circle one response.) ❑ more than 15% excluded ❑Not analysed as intention to treat

**Was the trial stopped early for benefit?** ❑Yes ❑No ❑Unclear

**Attrition Bias**

**Overall quality classification**

1. **For observational studies – New Castle Ottawa Quality Assessment Scale**

**Selection**

**Representativeness of the exposed cohort**

❑ Truly representative of the average adult in the comunity

❑ Somewhat representative of the average adult in the comunity

❑ Selected group of users

❑ No description

**Selection of the non exposed cohort**

❑ drawn from the same community as the exposed cohort

❑ drawn from a different source

❑ no description of the derivation of the non exposed cohort

**Ascertainment of exposure**

❑ secure record

❑ structured interview

❑ written self report

❑ no description

**Demonstration that outcomes of interest was not present at start of study**

❑ yes

❑ no

**Comparability**

**Comparability of cohorts on the basis of the design or analysis**

❑ study control for the most important factor ____________________

❑ study control for additional factors ___________________________

**Outcome**

**Assessment of outcome**

❑ independent blind assessment

❑ record linkage

❑ self report

❑ no description

**Was follow up long enough for outcomes to occur**

❑ yes

❑ no

**Adequacy of follow up of cohorts**

❑ all subjects

❑ subjects lost to follow up unlikely to introduce bias - < 5% lost to follow up

❑ subjects lost to follow up likely to introduce bias

❑ no statement

**5. Setting**

Where the study was conducted? (Country/countries) ________________________________

When was the study conducted? __________________________________________________

**4. Setting**

Where was the study conducted? (Country/countries) ________________________________

When was the study conducted? __________________________________________________

**Type of ICU(s)/ward** ❑Medical ❑Surgical ❑Medical/Surgical ❑Trauma/Neurosurgical ❑Rehabilitative ward

❑ Other, specify___________________________

**5. Participants**

| **Criterion** | **Total**  **____________________**  **(n= )** | **Group 1**  **__________________**  **(n= )** | **Group 2**  **________________**  **(n= )** |
| --- | --- | --- | --- |
| **N^O^ participants**  **(including dropouts)** |  |  |  |
| **N^O^ analyzed** |  |  |  |
| **Reasons for differences**  (if any) |  |  |  |
| **Inclusion criteria** |  | | |
| **Exclusion criteria** |  | | |
| **ASIA scale** | ❑A (No motor or sensory function preserved in S4-S5)  ❑B (Sensory but not motor function preserved below the neurologic level and includes S4-S5)  ❑C (motor function preserved below the neurologic level and more than half of the key muscles below the neurologic level have a muscle grade of < 3)  ❑D (motor function preserved below the neurologic level and at least half of the key muscles below the neurologic level have a muscle grade of > =3)  ❑E (motor and sensory function are normal) | | |
| **Level of the lesion** | ❑C _____________  ❑T _____________  ❑L _____________  ❑ Unknown | | |
| **Nature of the lesion** | ❑ Traumatic ❑Non-traumatic ❑Unknown | | |

**6. Study interventions**

| Is the mode of ventilator support used before weaning specified? | ❑Yes ❑Unclear ❑No |
| --- | --- |
| Is the used weaning technique described? | ❑Yes ❑Unclear ❑No |
| Was a weaning protocol used? | ❑Yes ❑Unclear ❑No |
| Was a rehabilitative program applied? | ❑Yes ❑Unclear ❑No |

**Did the authors provide weaning success/failure criteria?** ❑Yes ❑Unclear ❑No

**If yes, specify**

**______________________________________________________________________________________**

**Were extubation criteria reported?** ❑Yes ❑Unclear ❑No

**7. Study outcomes**

| Weaning outcome (success/failure) | ❑Yes ❑Unclear ❑No |
| --- | --- |
| Distinction among complete liberation from the ventilator, complete ventilator dependency and partial ventilator dependency | ❑Yes ❑Unclear ❑No |
| Reintubation rate | ❑Yes ❑Unclear ❑No |
| Time to partial or complete liberation from the ventilator | ❑Yes ❑Unclear ❑No |
| Factors associated with the ventilator outcomes | ❑Yes ❑Unclear ❑No |
| Total duration of ventilation | ❑Yes ❑Unclear ❑No |
| Number of weaning attempts | ❑Yes ❑Unclear ❑No |
| ICU length of stay | ❑Yes ❑Unclear ❑No |
| Hospital length of stay | ❑Yes ❑Unclear ❑No |
| Use of NIV following extubation | ❑Yes ❑Unclear ❑No |
| Mortality time point #1 __________  time point #2__________    time point #3__________ | ❑Yes ❑Unclear ❑No  ❑Yes ❑Unclear ❑No  ❑Yes ❑Unclear ❑No |
| Adverse event/complication related to MV   1. Tracheostomy 2. Prolonged MV at ____ days 3. Self-extubation 4. Other, specify______________________________________   _______________________________ | ❑Yes ❑Unclear ❑No |
| Other outcome  Specify,   1. _________________________ 2. _________________________ 3. _________________________ | ❑Yes ❑Unclear ❑No |

**8. Patients’ characteristics (continuous and dichotomous variables)**

| **Characteristics** | **Unit or relevant info** | **Total**  **(n = ) %** | **Group 1***  **(n = )** | **Group 2***  **(n= )**  **______________** | **95% CI or other information** |
| --- | --- | --- | --- | --- | --- |
| Age (mean SD/ median IQR) | y |  |  |  |  |
| Gender | Female |  |  |  |  |
| Neurological level of the lesion  (the most caudal segment with motor  and sensory function bilaterally) | Cervical  Thoracic  Lumbar |  |  |  |  |
| Completeness of the lesion | Complete  Incomplete |  |  |  |  |
| ASIA impairment scale | Grade A  Grade B  Grade C  Grade D  Grade E |  |  |  |  |
| Injury Severity Score (ISS) |  |  |  |  |  |
| **Characteristics** | **Unit or relevant info** | **Total**  **(n = ) %** | **Group 1***  **(n = )** | **Group 2***  **(n= )**  **______________** | **95% CI or other information** |
| Presence of additional extra spine injuries  Specify  _______________________ |  |  |  |  |  |
| Time before hospitalization in assessment hospital |  |  |  |  |  |
| Causes | Traumatic  Non-traumatic |  |  |  |  |
| Concomitant comorbidity | Obesity  COPD  Other relevant comorbidities |  |  |  |  |
| Other characteristic, Specify   1. _______________________ 2. _______________________ 3. _______________________ |  |  |  |  |  |

**9. Continuous outcomes**

| **Outcomes** | **Unit of measurement** | **Total** | | | **Group 1***  **_______________________** | | | **Group 2***  **__________________** | | | **95% CI or additional information** | |
| --- | --- | --- | --- | --- | --- | --- | --- | --- | --- | --- | --- | --- |
|  |  | **n** | **Mean**  **(SD)** | **Median (IQR)** | **n** | **Mean (SD)** | **Median (IQR)** | **n** | **Mean**  **(SD)** | **Median**  **(IQR)** | |  |
| Time to first SBT/weaning attempt |  |  |  |  |  |  |  |  |  |  | |  |
| Time to partial/complete liberation from the ventilator |  |  |  |  |  |  |  |  |  |  | |  |
| Total duration of weaning |  |  |  |  |  |  |  |  |  |  | |  |
| Number of weaning attempts |  |  |  |  |  |  |  |  |  |  | |  |
| Total duration of ventilation |  |  |  |  |  |  |  |  |  |  | |  |
| ICU length of stay |  |  |  |  |  |  |  |  |  |  | |  |
| Hospital length of stay |  |  |  |  |  |  |  |  |  |  | |  |
| Factors associated with the ventilator outcomes |  |  |  |  |  |  |  |  |  |  | |  |
| Other, please specify |  |  |  |  |  |  |  |  |  |  | |  |

**Continuous outcomes**

| **Outcomes** | **Unit of measurement** | **Total** | | | **Group 1***  **______________________** | | | **Group 2***  **_______________** | | | **95% CI or additional information** |
| --- | --- | --- | --- | --- | --- | --- | --- | --- | --- | --- | --- |
|  |  | **n** | **Mean**  **(SD)** | **Median (IQR)** | **n** | **Mean (SD)** | **Median (IQR)** | **n** | **Mean**  **(SD)** | **Median**  **(IQR)** |  |
| Time to first extubation attempt |  |  |  |  |  |  |  |  |  |  |  |
| Time to partial/complete liberation from the ventilator |  |  |  |  |  |  |  |  |  |  |  |
| Total duration of weaning |  |  |  |  |  |  |  |  |  |  |  |
| Number of weaning attempts |  |  |  |  |  |  |  |  |  |  |  |
| Total duration of ventilation |  |  |  |  |  |  |  |  |  |  |  |
| ICU length of stay |  |  |  |  |  |  |  |  |  |  |  |
| Hospital length of stay |  |  |  |  |  |  |  |  |  |  |  |
| Factors associated with the ventilator outcomes |  |  |  |  |  |  |  |  |  |  |  |
| Other, please specify |  |  |  |  |  |  |  |  |  |  |  |

**Continuous outcomes**

| **Outcomes** | **Unit of measurement** | **Total** | | | **Group 1***  **_______________________** | | | **Group 2***  **__________________** | | | **95% CI or additional information** |
| --- | --- | --- | --- | --- | --- | --- | --- | --- | --- | --- | --- |
|  |  | **n** | **Mean**  **(SD)** | **Median (IQR)** | **n** | **Mean (SD)** | **Median (IQR)** | **n** | **Mean**  **(SD)** | **Median**  **(IQR)** |  |
| Time to first extubation attempt |  |  |  |  |  |  |  |  |  |  |  |
| Time to partial/complete liberation from the ventilator |  |  |  |  |  |  |  |  |  |  |  |
| Total duration of weaning |  |  |  |  |  |  |  |  |  |  |  |
| Number of weaning attempts |  |  |  |  |  |  |  |  |  |  |  |
| Total duration of ventilation |  |  |  |  |  |  |  |  |  |  |  |
| ICU length of stay |  |  |  |  |  |  |  |  |  |  |  |
| Hospital length of stay |  |  |  |  |  |  |  |  |  |  |  |
| Factors associated with the ventilator outcomes |  |  |  |  |  |  |  |  |  |  |  |
| Other, please specify |  |  |  |  |  |  |  |  |  |  |  |

**Continuous outcomes**

| **Outcomes** | **Unit of measurement** | **Total** | | | **Group 1***  **_______________________** | | | **Group 2***  **__________________** | | | **95% CI or additional information** |
| --- | --- | --- | --- | --- | --- | --- | --- | --- | --- | --- | --- |
|  |  | **n** | **Mean**  **(SD)** | **Median (IQR)** | **n** | **Mean (SD)** | **Median (IQR)** | **n** | **Mean**  **(SD)** | **Median**  **(IQR)** |  |
| Time to first extubation attempt |  |  |  |  |  |  |  |  |  |  |  |
| Time to partial/complete liberation from the ventilator |  |  |  |  |  |  |  |  |  |  |  |
| Total duration of weaning |  |  |  |  |  |  |  |  |  |  |  |
| Number of weaning attempts |  |  |  |  |  |  |  |  |  |  |  |
| Total duration of ventilation |  |  |  |  |  |  |  |  |  |  |  |
| ICU length of stay |  |  |  |  |  |  |  |  |  |  |  |
| Hospital length of stay |  |  |  |  |  |  |  |  |  |  |  |
| Factors associated with the ventilator outcomes |  |  |  |  |  |  |  |  |  |  |  |
| Other, please specify |  |  |  |  |  |  |  |  |  |  |  |

**10. Dichotomous outcomes**

| **Outcomes** | **Total**  **(n = ) %** | **Group 1***  **(n = )** | **Group 2***  **(n= )**  **______________** | **95% CI or other information** |
| --- | --- | --- | --- | --- |
| Weaning success rate (complete liberation from the ventilator) |  |  |  |  |
| Rate of complete ventilator dependency |  |  |  |  |
| Rate of partial ventilator dependency |  |  |  |  |
| Number of weaning attempts |  |  |  |  |
| Mortality time point #1 |  |  |  |  |
| Mortality time point #2 |  |  |  |  |
| Mortality time point #3 |  |  |  |  |
| Use of non-invasive ventilation or pacing following extubation |  |  |  |  |
| Complications related to invasive MV |  |  |  |  |
| Adverse events:   - requirement for tracheostomy - self-extubation   other (specify) _________________________ |  |  |  |  |

**Dichotomous outcomes**

| **Outcomes** | **Total**  **(n = ) %** | **Group 1***  **(n = )** | **Group 2***  **(n= )**  **______________** | **95% CI or other information** |
| --- | --- | --- | --- | --- |
| Weaning success rate (complete liberation from the ventilator) |  |  |  |  |
| Rate of complete ventilator dependency |  |  |  |  |
| Rate of partial ventilator dependency |  |  |  |  |
| Number of weaning attempts |  |  |  |  |
| Mortality time point #1 |  |  |  |  |
| Mortality time point #2 |  |  |  |  |
| Mortality time point #3 |  |  |  |  |
| Use of non-invasive ventilation or pacing following extubation (specify) |  |  |  |  |
| Complications related to invasive MV |  |  |  |  |
| Adverse events:   - requirement for tracheostomy - self-extubation   other (specify) _________________________ |  |  |  |  |
| Other outcome, please specify |  |  |  |  |

**Dichotomous outcomes**

| **Outcomes** | **Total**  **(n = ) %** | **Group 1***  **(n = )** | **Group 2***  **(n= )**  **______________** | **95% CI or other information** |
| --- | --- | --- | --- | --- |
| Weaning success rate (complete liberation from the ventilator) |  |  |  |  |
| Rate of complete ventilator dependency |  |  |  |  |
| Rate of partial ventilator dependency |  |  |  |  |
| Number of weaning attempts |  |  |  |  |
| Mortality time point #1 |  |  |  |  |
| Mortality time point #2 |  |  |  |  |
| Mortality time point #3 |  |  |  |  |
| Use of non-invasive ventilation or pacing following extubation (specify) |  |  |  |  |
| Complications related to invasive MV |  |  |  |  |
| Adverse events:   - requirement for tracheostomy - self-extubation   other (specify) _________________________ |  |  |  |  |
| Other outcome, please specify |  |  |  |  |

**Dichotomous outcomes**

| **Outcomes** | **Total**  **(n = ) %** | **Group 1***  **(n = )** | **Group 2***  **(n= )**  **______________** | **95% CI or other information** |
| --- | --- | --- | --- | --- |
| Weaning success rate (complete liberation from the ventilator) |  |  |  |  |
| Rate of complete ventilator dependency |  |  |  |  |
| Rate of partial ventilator dependency |  |  |  |  |
| Number of weaning attempts |  |  |  |  |
| Mortality time point #1 |  |  |  |  |
| Mortality time point #2 |  |  |  |  |
| Mortality time point #3 |  |  |  |  |
| Use of non-invasive ventilation or pacing following extubation (specify) |  |  |  |  |
| Complications related to invasive MV |  |  |  |  |
| Adverse events:   - requirement for tracheostomy - self-extubation   other (specify) _________________________ |  |  |  |  |
| Other outcome, please specify |  |  |  |  |

* Specify Group 1 and Group 2

There can be different subgroups analyzed in the study:

- traumatic and non-traumatic
- level of the lesion affecting the diaphragm and not affecting the diaphragm
- complete lesion and incomplete
- cervical, thoracic or lumbar lesions
- additional extra spine injuries and no additional extra spine injuries

or interventions:

- weaning protocol and control group
- rehabilitation protocol and control group

**Please specify the numerator and denominator for each outcome.**

| **Other information which you feel is relevant to the results:**  Please provide data obtained from the primary author, additional results extrapolated from graphs, figures etc in the space provided below.  **Additional concerns/points to be clarified?** |
| --- |

**11. Risk of bias for outcomes in randomized controlled trials**

|  | Low | Moderate | High |
| --- | --- | --- | --- |
| Was randomization truly random? |  |  |  |
| Was allocation adequately concealed? |  |  |  |
| Were outcome assessors independent from individuals administering or supervising assigned interventions? |  |  |  |
| Were withdrawals described?  Did they occur with similar frequency between intervention and control groups |  |  |  |
| Were all patients analyzed according to the group they were initially assigned to whether they received it or not? |  |  |  |
| Was the trial stopped early for benefit?  What is the impact of early stopping of the trial, if applicable? |  |  |  |

Example: Allocation Concealment

*Low risk of bias:* central randomization (allocation by a central office unaware of subject characteristics); on-site computer system combined with allocation kept in a locked unreadable computer file that can be accessed only after the characteristics of an enrolled participant have been entered; sequentially numbered, sealed, opaque envelopes or other similar approaches that ensure the person who generated the allocation scheme did not administer it.

*Moderate risk of bias:* allocation concealment is unclear or when authors do not clearly report their approach.

*High risk of bias:* allocation concealment is not applied.

Overall assessment

❑ Low risk of bias: all criteria met

❑ Moderate risk of bias: one or more criteria unclear

❑ High risk of bias: one or more criteria not applied or met

**Complete statistical analysis**

For each study mean values and standard deviations (SDs) were preferred where available. In their absence, available measures including medians, ranges and 1^st^ – 3^rd^ quartile intervals (Q1-Q3) were used to estimate sample means and SDs (1, 2). For studies reporting values for subgroups only, the overall estimates were computed according to the Cochrane handbook guidelines (3).

The R package “meta” was used for all meta-analytic computations and plotting (4). Synthetic results are reported as meta-analytic means for continuous variables, and both as crude proportions and meta-analytic proportions, for categorical variables. To incorporate between-study heterogeneity (5), all analyses were performed using random effects models (6).

A 95% study confidence interval (95% CI) was evaluated for all characteristics and outcomes through the standard normal distribution for meta-analytic means and according to the Clopper-Pearson method for meta-proportions (7). Study weights were generated using the inverse variance method. Heterogeneity among studies was assessed by visual inspection of the forest plots, using the I^2^ statistic (threshold level for significant heterogeneity: ≥ 50%), Cochran’s Q and the Chi-squared test for homogeneity (significance level for heterogeneity: p < 0.1)(8).

Predefined subgroup analysis (significance level α = 0.05) was performed dividing studies conducted in ICUs vs rehabilitative settings.

In order to investigate the relationship between several covariates and the outcomes of weaning success and duration of mechanical ventilation, all predictors with available effect size were identified across the studies for both outcomes. Corresponding beta coefficients were pooled by weighting according to the inverse variance method (4). In order to obtain a robust estimate, univariate and multivariate regression model coefficients were pooled together (9).

Finally, we performed a post-hoc sensitivity analysis excluding the two largest studies (10, 11), as these studies enrolled patients from the United States National Trauma Databank in the period 2002-2006 and from the American College of Surgeons Trauma Quality Improvement Program database, potentially including patients reported in other included studies (12–19).

All statistical analyses were performed using R (version 4.0.3)(20).

**e-Table 1. Baseline characteristics of included studies**

| **Study author and**  **year** | **Country and**  **Inclusion period** | **Study design** | **No. of patients analyzed (enrolled) in each study*** | **Type of patients and**  **Spine lesion level**  **(level of anatomical lesion)** | **Type of ward and hospital** | **Mean age in years [95% CI]** | **Mean time from injury to hospitalization [95% CI]** |
| --- | --- | --- | --- | --- | --- | --- | --- |
| **ICU Studies** | | | | | | | |
| **Richard-Denis**  2018 | Canada  2008-2014 | Single-center retrospective cohort | 81 (81) | Traumatic  C1-C8 | Trauma-Neuro ICU  (Level I Trauma Center) | 42 [37.1 – 46.9] | 62.4 h [0 – 387.6] |
| **Call**  2011 | USA  2003-2007 | Single-center retrospective cohort | 80 (87) | Traumatic  C2-C7  T3-T12  L1-L5 | ICU not further specified  (Level I Trauma Center) | 39.3 [28.7 – 49.9] | NA |
| **Roquilly**  2014 | France  2001-2009 | Multi-center retrospective cohort | 164 (164) | Traumatic  C4-C6 | Surgical ICU | 43.3 [39.6 – 47] | 7.7 h [6.7 – 8.7] |
| **Kornblith**  2013 | USA  NA | Multi-center retrospective cohort | 344 (360) | Traumatic  C1-C7  T1-T12  L1-L5 | ICU not further specified  (Trauma Center) | 50.3 [44.6 – 56] | NA |
| **Fenton**  2015 | USA  NA | Single-center RCT | 33 (118) | Traumatic  C3-C6 | ICU not further specified  (Regional SCI Treatment Center) | 33.1 [29.1 – 37.1] | NA |
| **Como**  2005 | USA  2000-2002 | Single-center retrospective cohort | 45 (119) | Traumatic  C1-C6 | ICU not further specified  (Level I Trauma Center) | 40 [33.9 – 46.1] | 6.0 h [2.8 – 9.2] |
| **Flanagan**  2018 | USA  2007-2015 | Single-center retrospective cohort | 70 (80) | Traumatic  C2-C7 | ICU not further specified  (Level I Trauma Center) | 50.5 [45.8 – 55.2] | NA |
| **Yu**  2015 | Taiwan  2002-2012 | Single-center retrospective cohort | 73 (384) | Traumatic  C1-C7 | ICU not further specified  (Tertiary Medical Center) | 52.8 [48.4 – 57.2] | NA |
| **Liebscher**  2015 | Germany  2004-2010 | Single-center retrospective cohort | 37 (37) | Traumatic  C4-C8 | Trauma-Neuro ICU  (Spinal Cord Unit in Trauma Hospital) | 42.8 [36.2 – 49.4] | NA |
| **McCully**  2014 | USA  1998-2011 | Single-center retrospective cohort | 250 (250) | Traumatic  C1-T3 | ICU not further specified  (Level I Trauma Center) | 44.3 [41.3 – 47.3] | NA |
| **Choi**  2013 | Korea  2003-2012 | Single-center retrospective cohort | 21 (21) | Traumatic  C1-C7 | Trauma-Neuro ICU  (Neurosurgery Department, University Hospital) | 50 [26.2 – 73.8] | NA |
| **Leelapattana**  2012 | Canada  1991-2010 | Single-center retrospective cohort | 66 (66) | Traumatic  C4-C7 | Trauma-Neuro ICU  (Level I Trauma Center) | 37.6 [33.4 – 41.8] | NA |
| **Watt**  2011 | UK  1981-2005 | Single-center retrospective cohort | 189 (236) | Traumatic  C1-C8  T1-S5 | ICU not further specified  (Regional SCI Center) | NA | NA |
| **Ganuza**  2011 | Spain  2006-2008 | Single-center retrospective cohort | 323 (323) | Traumatic  C1-C8  T1-T12 | Polyvalent ICU  (Dedicated SCI Hospital) | 42.3 [ 40.8 – 43.8] | NA |
| **Branco**  2011 | USA  2002-2006 | Multi-center retrospective cohort | 5256 (5256) | Traumatic  C1-C7 | NA (probably mainly ICUs)  National Trauma databank | 43.3 [42.7 – 43.9] | NA |
| **Romero**  2009 | Spain  2004-2007 | Single-center retrospective cohort | 152 (164) | Traumatic  C3-C8  T1-T12 | Trauma-Neuro ICU  (SCI Hospital) | 41.1 [40.6 – 41.6] | NA |
| **Higashi**  2019 | Japan  2010-2017 | Single-center retrospective cohort | 65 (65) | Traumatic  C2-C8  T1 | ICU not further specified (Advanced Critical Care & Emergency Center) | 72.8 [70.8 – 74.8]^#^ | NA |
| **Alizo**  2018 | USA  2011-2015 | Single-center quality improvement study | 95 (95) | Traumatic  Cervical + others not further specified | ICU not further specified  (Level II Trauma Center) | 46.9 [43.1 – 50.7] | NA |
| **Jones**  2015 | USA  1998-2012 | Single-center retrospective cohort | 163 (163) | Traumatic  C1-C8 | ICU not further specified  (Level I Regional Trauma Center) | 39 [36.4 – 41.6] | NA |
| **Raurich**  2014 | Spain  2005-2009 | Multi-center retrospective cohort | 12 (43) | Not specified  C4-C7 | Medical-Surgical ICU  (University Hospital) | 26 [22 – 30] | NA |
| **Gardner**  1986 | UK  1969-1984 | Single-center retrospective cohort | 44 (44) | Not specified  C2-C7  T3-L1 | ICU not further specified  (Regional Spinal Injury Center) | 37.9 [32.7 – 43.1] | NA |
| **Beom**  2018 | Korea  2005-2016 | Single-center retrospective cohort | 49 (49) | Traumatic  C1-C7 | ICU not further specified  (University Hospital) | NA | NA |
| **Anand**  2020 | USA  2010-2014 | Multi-center retrospective cohort | 5980 (5980) | Traumatic  C1-C7 | NA (probably mainly ICUs)  American College Surgeons Trauma Quality Improvement database | 46.7 [46.1 – 47.3] | NA |
| **Shah**  2021 | Nepal  2017-2018 | Single-center retrospective cohort | 48 (48) | Traumatic  C1-C7 | ICU not further specified  (Tertiary Referral Trauma Center) | 43.9 [39.4 – 48.4] | NA |
| **Cinotti**  2019 | France  2001-2016 | Single-center retrospective cohort | 117 (117) | Traumatic  C1-T1 | Surgical ICU  (University Hospital) | 46.5 [42.5 –50.5] | 8.3 h [6.8 – 9.8] |
| **Rehabilitative Setting Studies** | | | | | | | |
| **Gundogdu**  2016 | Turkey  2012-2015 | Single-center prospective  observational trial | 10 (35) | Traumatic  C1-C6 | Rehabilitation ward  (Physical Medicine and Rehabilitation Clinic Tertiary Hospital) | 27.1 [16.9 – 37.3] | 57.4 days [33.0 – 81.8] |
| **Füssenich**  2018 | Germany  2010-2017 | Single-center retrospective cohort | 165 (165) | Traumatic and non-traumatic  C1-C8  T1-S5 | ICU and Intermediate Care Unit  (SCI Department Level I Trauma Center) | 57.2 [54.6 – 59.8] | 24.0 days [20.9 – 27.1] |
| **Chiodo**  2008 | USA  NA | Single-center retrospective cohort | 29 (29) | Not specified  C2-C6 | Rehabilitation ward  (SCI Rehabilitation Unit University Hospital) | 43 [35.7 – 50.3] | 71.0 days [49.5 – 92.3] |
| **Zakrasek**  2017 | USA  2013-2014 | Single-center retrospective cohort | 36 (36) | Traumatic  C1-C5 | Rehabilitation ward  (SCI/Trauma Center) | NA | 28.0 days [23.2 – 32.8] |
| **Kim**  2018 | Korea  NA | Single-center retrospective cohort | 67 (67) | Not specified  C2-C5 | Rehabilitation ward (Department of Rehabilitation Medicine, University Hospital) | 56.6 [52.8 – 60.4] | 37.0 days [0.5 – 73.5] |
| **Atito-Narh**  2008 | UK  1995-2006 | Single-center retrospective cohort | 13 (13) | Traumatic  C2-C6 | Rehabilitation ward  (Regional SCI Center) | 43.8 [34 – 53.6] | 102.0 days [61.2 – 142.8] |
| **Peterson**  1999 | USA  1983-1993 | Single-center retrospective cohort | 42 (42) | Not specified  C3-C4 | Rehabilitation ward (Regional SCI Center) | 30 [26.2 – 33.8] | 52.7 days [45.4 – 60.0] |
| **Wicks**  1986 | USA  1974-1983 | Single-center retrospective cohort | 134 (134) | Traumatic  C1-C8 | Subacute Neuro-Trauma Unit  (Regional SCI Center) | NA | NA |
| **Toki**  2019 | Japan  2005-2010 | Single-center retrospective cohort | 14 (14) | Not specified  C1-C3 | Rehabilitation ward  (Public Hospital) | 31 [23.8 – 38.2] | 378 days [0 – 1456.0] |
| **Kim**  2017 | Korea  2003-2015 | Single-center retrospective cohort | 43 (62) | Not specified  C1-C8 | Rehabilitation ward  (Pulmonary rehabilitation center, Tertiary University Hospital) | NA | NA |
| **Wong**  2012 | USA  2010-2011 | Single-center retrospective cohort | 24 (24) | Traumatic and non-traumatic  C1-C4 | Rehabilitation ward  (SCI Specialized Center) | 33.4 [26.8 – 40] | 33.8 days [24.0 – 43.6] |
| **Wallbom**  2005 | USA  1998-2002 | Single-center retrospective cohort | 33 (68) | Not specified  C2-C4 | Rehabilitation ward  (Rehabilitation Trauma Center) | NA | 9.0 days [5.2 – 12.8] |
| **Bach**  1991 | USA  1980-1988 | Single-center retrospective cohort | 34 (38) | Traumatic  Quadriplegic (no further specification on the level of the lesion) | Rehabilitation ward (Pulmonary Rehabilitation Unit, University Hospital) | 29.4 [23.4 – 35.4] | NA |
| **Fuhrer**  1987 | USA  1975-1981 | Multi-center retrospective cohort | 230 (230) | Traumatic  C1-C4 | Rehabilitation wards (National SCI Database) | NA | NA |

White rows indicate studies in ICU, shaded rows indicate studies performed in rehabilitative setting.

* Number of patients whose data were analyzed in each study. In brackets total number of participants of each study.

^#^ Patients under 60 years of age were excluded as per study design.

**e-Table 2. Baseline characteristics of included studies II**

| **Study author and**  **year** | **Inclusion criteria** | **Exclusion criteria** | **Outcomes of interest assessed** | **Presence of regression analysis** |
| --- | --- | --- | --- | --- |
| **ICU Studies** | | | | |
| **Richard-Denis**  2018 | - traumatic SCI  - cervical lesion  - treated surgically | - managed not surgically | - Weaning success  - Duration of mechanical ventilation  - Tracheostomy  - ICU length of stay  - Hospital length of stay  - Mortality | Univariate and Multivariate |
| **Call**  2011 | - SCI mechanically ventilated | NA | - Weaning success  - Tracheostomy  - ICU length of stay  - Hospital length of stay  - Pneumonia  - Mortality | NA |
| **Roquilly**  2014 | - adult patients  - hospitalized for acute (≤ 24 h) traumatic tetraplegia | - > 24 h between injury and ICU admission  - non-traumatic  - traumatic brain injury  - death within 48 h of ICU admission | - Duration of mechanical ventilation  - ICU length of stay  - Pneumonia  - Mortality | Univariate and Multivariate |
| **Kornblith**  2013 | - traumatic SCI  - requiring mechanical ventilation | NA | - Weaning success  - Tracheostomy  - ICU length of stay  - Hospital length of stay  - Pneumonia  - Mortality | Multivariate |
| **Fenton**  2015 | - adult (18-55)  - traumatic SCI  - cervical lesion (C3-C6)  - ASIA A/B/C  - > 2 weeks and within 6 months from injury  - ventilator dependent (on continuous mechanical ventilation) | - concurrent traumatic brain injury/chest trauma/critical illness polyneuropathy  - chronic obstructive pulmonary disease  - Body mass index > 35  - diaphragm paralysis | - Duration of mechanical ventilation  - Pneumonia | NA |
| **Como**  2005 | - cervical SCI  - neurologic deficit | NA | - Weaning success  - Tracheostomy  - ICU length of stay  - Mortality | NA |
| **Flanagan**  2018 | - traumatic SCI  - tracheostomized during the initial stay | - prior SCI  - prior tracheostomy for any indication  - laryngeal cancer  - insufficient available data in the medical records | - Duration of mechanical ventilation  - ICU length of stay  - Tracheostomy  - Pneumonia  - Mortality | Univariate and Multivariate |
| **Yu**  2015 | - traumatic SCI  - cervical lesion | - > 2 weeks between injury and admission  - < 18 years  - medical charts unavailable  - mechanical ventilation for < 48 h for surgical requirement  - transferred to another hospital for a reason different than weaning failure  - died before successful weaning without completing the weaning protocol | - Weaning success  - Duration of mechanical ventilation  - ICU length of stay  - Hospital length of stay  - Pneumonia | Univariate and Multivariate |
| **Liebscher**  2015 | - acute isolated traumatic SCI  - cervical lesion (C4-C8)  - complete motor function loss  - indication for spinal surgery | - multiple spinal cord lesions  - central cord syndrome/Brown-Séquard syndrome/anterior cord syndrome  - head injury  - cerebral stroke  - presence of any unstable medical or psychiatric conditions | - Weaning success  - Duration of mechanical ventilation  - Tracheostomy  - Decannulation  - ICU length of stay  - Hospital length of stay  - Pneumonia  - Mortality | Univariate and Multivariate |
| **McCully**  2014 | - traumatic SCI  - cervical and thoracic lesion (C1-T3) | - traumatic brain injury  - death within 24 h | - Duration of mechanical ventilation  - Tracheostomy  - ICU length of stay  - Hospital length of stay | Univariate and Multivariate |
| **Choi**  2013 | - traumatic SCI | - degenerative spinal disease/spinal tumors/inflammatory disease of the spine | - Duration of mechanical ventilation  - Tracheostomy  - ICU length of stay  - Hospital length of stay  - Pneumonia | Univariate |
| **Leelapattana**  2012 | - ≥ 16 years of age  - cervical lesion (C4-C7)  - admission to hospital within 24 h from injury  - > 24 h of mechanical ventilation | - death within 7 days from injury  - postoperative mechanical ventilation (for < 24 h) | - Duration of mechanical ventilation  - Tracheostomy  - ICU length of stay  - Hospital length of stay  - Pneumonia  - Mortality | Univariate and Multivariate |
| **Watt**  2011 | - adult and children  - traumatic SCI  - needing mechanical ventilation on their first admission | - non-traumatic SCI  - mechanical ventilation for < 5 days | - Weaning success  - Hospital length of stay  - Mortality | NA |
| **Ganuza**  2011 | - recent traumatic SCI  - cervical or thoracic lesion  - admitted to the ICU during their first inpatient episode | - history of emergency tracheostomy/previous tracheostomy/prior airway problems  - coagulopathies | - Tracheostomy  - Pneumonia  - Mortality | Multivariate |
| **Branco**  2011 | - traumatic SCI  - cervical lesion | - traumatic brain injury | - Duration of mechanical ventilation  - Tracheostomy  - ICU length of stay  - Hospital length of stay  - Mortality | Univariate and Multivariate |
| **Romero**  2009 | - new traumatic SCI  - acute/subacute stage admitted to the ICU | - cervical spine lesion above C3 requiring permanent mechanical ventilation  - missing data  - comprised airway | - Duration of mechanical ventilation  - Tracheostomy  - ICU length of stay  - Pneumonia  - Mortality | Multivariate |
| **Higashi**  2019 | - traumatic SCI | - < 60 years of age  - severe head injury (traumatic brain injury) | - Tracheostomy  - Decannulation  - ICU length of stay  - Mortality | Univariate and Multivariate |
| **Alizo**  2018 | - traumatic SCI | - non-traumatic SCI  - cerebral vascular accident-related deficits  - trauma patients no neurologic deficits  - no radiographic evidence of SCI | - Duration of mechanical ventilation  - ICU length of stay  - Hospital length of stay  - Pneumonia  - Mortality | NA |
| **Jones**  2015 | - traumatic SCI  - cervical lesion | - death within 48 h of admission | - Weaning success  - Tracheostomy  - Decannulation  - ICU length of stay  - Hospital length of stay  - Pneumonia  - Mortality | NA |
| **Raurich**  2014 | - acute SCI  - cervical lesion  - tetraplegia (complete lesion, ASIA A)  - needing mechanical ventilation | - concomitant head/thoracic/abdominal injuries | - Weaning success  - Duration of mechanical ventilation  - Tracheostomy  - ICU length of stay  - Hospital length of stay  - Mortality | NA |
| **Gardner**  1986 | - SCI  - ventilated | NA | - Weaning success  (complete vs partial)  - Mortality | NA |
| **Beom**  2018 | - traumatic SCI  - cervical lesion  - ASIA < 50  - who underwent surgery for cervical spine injury | NA | - Duration of mechanical ventilation*  - Tracheostomy  - ICU length of stay * | NA |
| **Anand**  2020 | - ≥ 18 years of age  - traumatic SCI  - cervical lesion  - tracheostomized | - transferred from other institution  - missing data about SCI variables, Injury Severity Score, tracheostomy | - Tracheostomy  - ICU length of stay  - Mortality | Univariate and Multivariate |
| **Shah**  2021 | - traumatic SCI  - cervical lesion | - spondylodiscitis  - tumors  - history of previous spine surgery | - Tracheostomy  - ICU length of stay  - Mortality | NA |
| **Cinotti**  2019 | - ≥ 18 years of age  - traumatic SCI  - lesion T1 or above | - concomitant traumatic brain injury/ Glasgow Coma Scale ≤ 12  - admission to ICU ≥ 48 h after trauma  - non-traumatic SCI | - Duration of mechanical ventilation  - Tracheostomy  - ICU length of stay  - Pneumonia  - Mortality | Multivariate |
| **Rehabilitative Setting Studies** | | | | |
| **Gundogdu**  2016 | - traumatic SCI  - cervical lesion  - ASIA A/B/C  - mechanically ventilated/tracheostomized | - requiring invasive monitoring | - Weaning success  - Duration of mechanical ventilation  - Tracheostomy  - Decannulation  - Rehab length of stay  - Pneumonia | NA |
| **Füssenich**  2018 | - traumatic and non-traumatic SCI | - < 18 years of age  - no diaphragm function  - with central sleep disorder | - Weaning success  - Pneumonia | NA |
| **Chiodo**  2008 | - ventilator dependent SCI  - admitted to acute rehabilitation | NA | - Weaning success  - Duration of mechanical ventilation  - Pneumonia | NA |
| **Zakrasek**  2017 | - traumatic SCI  - cervical lesion (C1-C5)  - ASIA A/B  - within 3 months from injury  - tracheostomized and ventilator dependent | - ventilator free breathing for > 16 h at the time of admission | - Weaning success (complete vs partial)  - Duration of mechanical ventilation  - Tracheostomy  - Decannulation | Univariate |
| **Kim**  2018 | - SCI  - cervical lesion (C2-5)  - w/o ventilator need at the time of admission | - haemodynamic instability  - cognitive impairment/sedative state  - spinal shock | - Weaning success  - Duration of mechanical ventilation  - Rehab length of stay | NA |
| **Atito-Narh**  2008 | - history of failed weaning  - duration of mechanical ventilation > 21 days | NA | - Weaning success  - Duration of mechanical ventilation  - Tracheostomy  - Decannulation  - Pneumonia  - Mortality | NA |
| **Peterson**  1999 | - ventilator dependency (24h/day mechanical ventilation)  - weaned at discharge | NA | - Weaning success  - Duration of mechanical ventilation  - Tracheostomy  - Pneumonia | NA |
| **Wicks**  1986 | - traumatic quadriplegia on continuous mechanical ventilation for > 30 days,  - injury < 365 days prior to admission  - first hospital admission | NA | - Weaning success  - Mortality | NA |
| **Toki**  2019 | - tracheostomized ventilator-dependent patients  - cervical lesion (C1-C3)  - ASIA A | - swallowing disorders  - poor comprehension/cooperation  - poor ventilatory status | - Weaning success (complete vs partial)  - Tracheostomy  - Decannulation  - Pneumonia  - Mortality | NA |
| **Kim**  2017 | NA | NA | - Weaning success  (complete vs partial) | NA |
| **Wong**  2012 | - SCI  - cervical lesion (C1-C4)  - ventilated | NA | - Weaning success (complete vs partial)  - Duration of mechanical ventilation  - Tracheostomy  - Decannulation  - Pneumonia | NA |
| **Wallbom**  2005 | - SCI  - cervical lesion (C1-C4) and tetraplegia  - patients who underwent acute rehabilitation | - patients who did not receive acute inpatient rehabilitation at their center | - Weaning success  - Duration of mechanical ventilation  - Tracheostomy  - Decannulation | NA |
| **Bach**  1991 | - traumatic SCI  - quadriplegic  - ventilator-dependent - referred to pulmonary rehabilitation | - uncooperative patients  - with severe complicating medical conditions/cognitive deficits | - Weaning success (complete vs partial)  - Tracheostomy  - Decannulation  - Mortality | NA |
| **Fuhrer**  1987 | - SCI  - cervical lesion (C1-C4)  - who received mechanical ventilation either during acute care/rehabilitation  - discharged from inpatient rehabilitation  - had a follow-up 1 year post-injury | NA | - Weaning success  - Mortality | NA |

White rows indicate studies pin ICU, shaded rows indicate studies performed in rehabilitative setting.

Abbreviations: ASIA= American Spinal Injury Association, SCI= spinal cord injury, ICU= intensive care unit.

* Duration of mechanical ventilation and ICU length of stay were assessed in the study; however, data from these outcomes were not usable due to absent reporting of uncertainty measures (namely, standard deviations, ranges, 1^st^ – 3^rd^ quartile intervals, etc.).

**e-Table 3. The Newcastle-Ottawa Scale for assessing the quality of nonrandomized studies**

|  | **Selection** | | | **Comparability** | **Outcome** | | |  |
| --- | --- | --- | --- | --- | --- | --- | --- | --- |
| **Study** | **Representativeness**  **of the exposed cohort** | **Ascertainment of exposure** | **Outcome of interest was not present at start of study** | **Comparability of cohorts on the basis of the design or analysis** | **Assessment of outcome** | **Follow-up long enough for outcomes to occur** | **Adequacy of follow up of cohorts** | **Total score**  **(8)** |
| **Richard-Denis**  2018 | * | * | * | ** | * | * | * | 8/8 |
| **Call**  2011 | * | * | * | - | * | * | * | 7/8 |
| **Roquilly**  2014 | - | * | * | ** | * | * | * | 7/8 |
| **Kornblith**  2013 | * | * | * | ** | * | * | * | 8/8 |
| **Como**  2005 | * | * | * | - | * | * | * | 6/8 |
| **Flanagan**  2018 | * | * | * | ** | * | * | * | 8/8 |
| **Yu**  2015 | - | * | * | ** | * | * | * | 7/8 |
| **Liebscher**  2015 | - | * | * | ** | - | * | * | 6/8 |
| **McCully**  2014 | - | * | * | ** | * | * | * | 7/8 |
| **Choi**  2013 | * | * | * | * | * | * | * | 7/8 |
| **Leelapattana**  2012 | - | * | * | ** | * | * | * | 7/8 |
| **Watt**  2011 | * | * | * | - | - | * | * | 5/8 |
| **Ganuza**  2011 | * | * | * | ** | * | * | * | 8/8 |
| **Branco**  2011 | - | * | * | ** | * | * | * | 7/8 |
| **Romero**  2009 | - | * | * | ** | * | * | * | 7/8 |
| **Higashi**  2019 | - | * | * | ** | * | * | * | 7/8 |
| **Alizo**  2018 | * | * | * | - | * | * | * | 6/8 |
| **Jones**  2015 | - | - | * | - | * | * | * | 4/8 |
| **Raurich**  2014 | - | * | * | - | * | * | * | 5/8 |
| **Gardner**  1986 | * | - | * | - | - | * | * | 4/8 |
| **Beom**  2018 | * | - | * | - | * | * | * | 5/8 |
| **Anand**  2020 | * | * | * | * | * | * | * | 7/8 |
| **Shah**  2021 | - | - | * | - | * | * | * | 4/8 |
| **Cinotti**  2019 | * | * | * | * | * | * | * | 7/8 |
| **Gundogdu**  2016 | * | * | * | - | * | * | * | 6/8 |
| **Füssenich**  2018 | * | * | * | - | * | * | * | 6/8 |
| **Chiodo**  2008 | * | * | * | - | * | * | * | 6/8 |
| **Zakrasek**  2017 | * | * | * | * | * | * | * | 7/8 |
| **Kim**  2018 | * | - | * | - | * | * | * | 6/8 |
| **Atito-Narh**  2008 | - | * | * | - | * | * | * | 5/8 |
| **Peterson**  1999 | - | * | * | - | * | * | * | 5/8 |
| **Wicks**  1986 | - | * | * | - | * | * | * | 5/8 |
| **Toki**  2019 | - | - | * | - | * | * | * | 4/8 |
| **Kim**  2017 | - | * | * | - | * | * | * | 5/8 |
| **Wong**  2012 | - | - | * | - | * | * | * | 4/8 |
| **Wallbom**  2005 | - | * | * | - | * | * | * | 5/8 |
| **Bach**  1991 | * | - | * | - | * | * | * | 5/8 |
| **Fuhrer**  1987 | - | - | * | - | - | * | * | 3/8 |

A study can be awarded a maximum of three stars in the Selection category, a maximum of two stars in the Comparability category, and a maximum of three stars in the Outcome category. The number of stars indicates the methodologic quality of the study. Fewer stars correspond with lower quality.

**e-Table 4. Baseline characteristics of the patients enrolled in each study**

| **Characteristics at baseline** | **Means**  **and**  **Proportions** | **Overall**  (n= 14637) | **ICU**  (n= 13763) | **Rehab Unit**  (n= 874) | | **Number of Studies, total (ICU + rehab}** | **p-value^#^** | |
| --- | --- | --- | --- | --- | --- | --- | --- | --- |
| Age, years | Meta-analytic | 42.9 [40.5 – 45.3] | 44.0 [41.4 – 46.6] | | 39.2 [29.8 – 48.7] | 31 (22 + 9) | 0.3427 | |
| Gender, male, n (%) | Crude | 11076/14498 (76.4%) | 10457/13763 (76.0%) | | 619/735 (84.2%) | 34 (24 + 10) |  | |
|  | Meta-analytic | 80.2% [78.1% – 82.1%] | 78.6% [76.5% – 80.6%] | | 84.2% [81.2% – 86.9%] |  | **0.0029** | |
| Comorbidities, n (%) |  |  |  | |  |  |  | |
| *Respiratory comorbidities* | Crude | 664/6583 (10.1%) | 643/6365 (10.1%) | | 21/218 (9.6%) | 9 (6 + 3) |  |  |
|  | Meta-analytic | 6.3% [3.7% – 10.7%] | 5.1% [2.6% – 9.6%] | | 10.4% [4.9% – 20.6%] |  | 0.1488 |  |
| *Cardiovascular comorbidities* | Crude | 267/6377 (4.2%) | 261/6324 (4.1%) | | 6/53 (11.3) | 7 (5 + 2) |  |  |
|  | Meta-analytic | 11.4% [4.9% – 24.2%] | 12.4% [4.3% – 30.5%] | | 11.1% [4.3% – 25.5%] |  | 0.8729 |  |
| *Other comorbidities* | Crude | 733/6695 (10.9%) | 620/6441 (9.6%) | | 113/254 (44.5%) | 8 (6 + 2) |  | |
|  | Meta-analytic | 16.3% [8.0% – 30.2%] | 15.0% [7.4% – 28.0%] | | 18.9% [2.3% – 69.8%] |  | 0.8204 | |
| Glasgow Coma Scale on admission | Meta-analytic | 11.8 [10.7 – 12.8] | 11.8 [10.7 – 12.8] | | - | 10 (10 + 0) | - | |
| Time from injury to admission | Meta-analytic |  | 7.8 hours [7.0 – 8.6] | | 39.9 days [28.6 – 51.3] | 14 (4 + 10) | **<0.0001** | |
| **Characteristics of the lesion** |  |  |  |  | |  |  | |
| Blunt as primary injury type, n (%) | Crude | 11115/12542 (88.6%) | 11115/12542 (88.6%) | - | | 8 (8 + 0) | - | |
|  | Meta-analytic | 83.3% [60.7% – 94.1%] | 83.3% [60.7% – 94.1%] | - | |  |  | |
| Mechanism of injury, n (%) |  |  |  |  | |  |  | |
| *Falls* | Crude | 447/1258 (35.5%) | 389/1041 (37.4%) | 58/217 (26.7%) | | 14 (9 + 5) |  | |
|  | Meta-analytic | 38.3% [28.0% – 49.7%] | 43.1% [28.7% – 58.9%] | 26.7% [21.3% – 33.0%] | |  | 0.0414 | |
| *Transport* | Crude | 597/1258 (47.5%) | 494/1041 (47.5%) | 103/217 (47.5%) | | 14 (9 + 5) |  | |
|  | Meta-analytic | 40.3% [30.2% – 51.4%] | 39.0% [25.0% – 55.2%]1 | 47.5% [40.9% – 54.1%] | |  | 0.3351 | |
| *Assault* | Crude | 27/1258 (2.1%) | 4/1041 (0.4%) | 23/217 (10.6%) | | 14 (9 + 5) |  | |
|  | Meta-analytic | 3.4% [1.0% – 10.6%] | 0.8% [0.3% – 2.0%] | 11.1% [6.6% – 17.9%] | |  | **<0.0001** | |
| *Sports* | Crude | 74/1258 (5.9%) | 52/1041 (5.0%) | 22/217 (10.1%) | | 14 (9 + 5) |  | |
|  | Meta-analytic | 10.8% [5.9% – 18.9%] | 7.7% [1.0% – 41.3%] | 10.1% [6.8% – 14.9%] | |  | 0.7818 | |
| *Other* | Crude | 50/1258 (4.0%) | 39/1041 (3.7%) | 11/217 (5.1%) | | 14 (9 + 5) |  | |
|  | Meta-analytic | 5.1% [3.2% – 8.1%] | 5.6% [3.2% – 8.10%] | 5.1% [2.8% – 8.9%] | |  | 0.7968 | |
| Level of the spinal lesion, n |  |  |  |  | |  |  | |
| *Cervical spine lesion only* |  | 12717 |  |  | |  |  | |
| *Cervical spine lesion + others* |  | 1843 |  |  | |  |  | |
| *Cervical* |  | 1365 |  |  | |  |  | |
| *Thoracic* |  | 383 |  |  | |  |  | |
| *Lumbar* |  | 51 |  |  | |  |  | |
| *Unknown* |  | 44 |  |  | |  |  | |
| *Unknown level* |  | 77 |  |  | |  |  | |
| *C3 or above* | Crude | 5375/13047 (41.2%) | 5052/12387 (40.8%) | | 323/660 (48.9%) | 27 (18 + 9) |  | |
|  | Meta-analytic | 25.4% [14.1% – 41.4%] | 14.1% [6.1% – 29.1%] | | 55.1% [40.2% – 69.2%] |  | **0.0003** | |
| *C4 or below* | Crude | 8033/13047 (61.6%) | 7696/12387 (62.1%) | | 337/660 (51.1%) | 27 (18 + 9) |  | |
|  | Meta-analytic | 74.6% [58.7% – 85.8%] | 85.9% [71.0% – 93.8%] | | 44.9% [30.8% – 59.8%] |  | **0.0003** | |
| Complete syndrome, n (%) | Crude | 3761/13028 (28.9%) | 3537/12685 (27.9%) | | 224/343 (65.3%) | 17 (13 + 4) |  | |
|  | Meta-analytic | 69.8% [49.0% – 84.8%] | 71.3% [41.9% – 89.6%] | | 68.5% [53.7% – 80.3%] |  | 0.8516 | |
| Extra spine lesions, n (%) | Crude | 2661/6647 (40.0%) | 2661/6647 (40.0%) | | - | 14 (14 + 0) |  | |
|  | Meta-analytic | 20.7% [9.8% – 38.6%] | 20.7% [9.8% – 38.6%] | | - |  | - | |
| Injury Severity Score | Crude | 26.3 [23.6 – 29.0] | 26.3 [23.6 – 29.0] | | - | 13 (13 + 0) |  | |

Meta-analytic means and proportions were computed through the “metamean” and ”metaprop” functions (R package “meta”), respectively

^#^ P-values yielded by the subgroup analyses: ICUs vs Rehabilitation Units.

**e-Table 5. Summary of definitions: complete weaning, partial weaning**

| **Complete weaning** | |
| --- | --- |
| **Gundogdu** 2016 | ≥ 2 weeks of ventilator free breathing |
| **Füssenich** 2018 | ≥ 7 days of ventilator free breathing |
| **Fenton** 2015 | 72 h of ventilator free breathing |
| **Zakrasek** 2017 | 24 h of ventilator free breathing |
| **Chiodo** 2008 **Peterson** 1999  **Toki** 2019  **Kim** 2017 | Ventilator free breathing, including night-time (not specified for how long) |
| **Yu** 2015  **Fuhrer** 1987 | Ventilator free breathing at discharge |
| **Richard-Denis** 2018  **Como** 2005 **Liebscher** 2015 **Watt** 2011  **Jones** 2015  **Raurich** 2014 **Gardner** 1986  **Kim** 2018  **Atito-Narh** 2008 **Wicks** 1986  **Wong** 2012 **Wallbom** 2005 **Bach** 1991  **Fuhrer** 1987 | No definition |
| **Partial weaning** | |
| **Zakrasek** 2017 | 16 h of ventilator free breathing |
| **Gardner** 1986 | Ventilated overnight |
| **Toki** 2019  **Kim** 2017  **Wong** 2012  **Bach** 1991 | Use on non-invasive ventilation (+/- iron lung for overnight support) |
| **Wicks** 1986 | No definition |

**e-Table 6. Predictors for the outcome of weaning failure.** Logistic regression analyses across the studies

| **Variable** | **Pooled beta** | **95% Confidence Interval** | **p-value** | **Number of pooled estimates** |
| --- | --- | --- | --- | --- |
| Age | -0.01 | [-0.02 – 0.01] | 0.2575 | 4 |
| Gender (ref= male) | -0.01 | [-0.54 – 0.52] | 0.9598 | 2 |
| Body mass index | 0.003 | [-0.03 – 0.03] | 0.8455 | 3 |
| Diabetes | 0.97 | [-0.25 – 2.20] | 0.118 | 1 |
| Respiratory comorbidities | 0.12 | [-0.48 – 0.73] | 0.6887 | 2 |
| Number of comorbidities | 0.79 | [1.39 – 0.19] | **0.0104** | 2 |
| Blunt | -0.05 | [-1.02 – 0.92] | 0.9192 | 2 |
| Injury Severity Score | 0.04 | [0.03 – 0.06] | **<0.0001** | 2 |
| Thoracic Abbreviated Injury Scale (AIS) | -0.04 | [-0.26 – 0.17] | 0.6835 | 2 |
| Head Abbreviated Injury Scale (AIS) | 0.16 | [-0.27 – 0.60] | 0.4647 | 2 |
| Traumatic Brain Injury | 0.56 | [-0.06 – 1.18] | 0.0745 | 2 |
| Other injury | -0.12 | [-0.85 – 0.61] | 0.7495 | 2 |
| C1-C3 SCI level (ref= C4-C7) | 2.31 | [1.26 – 3.35] | **<0.0001** | 2 |
| Glasgow Coma Scale on admission | 0.01 | [-0.05 – 0.07] | 0.6743 | 3 |
| Respiratory failure on admission | 0.84 | [-0.24 – 1.91] | 0.127 | 1 |
| Heart rate on admission | 0.05 | [0.01 – 0.08] | **0.0085** | 2 |
| Mean blood pressure on admission | 0.02 | [-0.01 – 0.05] | 0.13 | 1 |
| Respiratory rate at intubation | 0.01 | [-0.03 – 0.06] | 0.5678 | 3 |
| pH at intubation | -0.52 | [-3.32 – 2.29] | 0.717 | 2 |
| pCO_2_ at intubation | -0.01 | [-0.04 – 0.02] | 0.4459 | 2 |
| HCO_3_ at intubation | -0.04 | [-0.13 – 0.06] | 0.45 | 2 |
| Base deficit at intubation | -0.02 | [-0.09 – 0.04] | 0.5119 | 2 |
| FiO_2_ at intubation | 0.73 | [-0.15 – 1.60] | 0.1035 | 2 |
| Minute ventilation at intubation | -0.10 | [-0.28 – 0.09] | 0.3143 | 2 |
| Peak pressure at intubation | 0.03 | [-0.02 – 0.07] | 0.2749 | 2 |
| Plateau pressure at intubation | 0.08 | [-0.01 – 0.16] | 0.073 | 2 |
| Positive end-expiratory pressure at intubation | -0.15 | [-0.34 – 0.03] | 0.1093 | 2 |
| SpO_2_ at intubation | 0.06 | [-0.01 – 0.13] | 0.0853 | 2 |
| Mean arterial pressure at intubation | -0.01 | [-0.05 – 0.03] | 0.527 | 2 |
| Tracheostomy | 2.81 | [1.86 – 3.77] | **<0.0001** | 2 |

Corresponding beta coefficients were pooled by weighting according to the inverse variance as implemented by the “metagen” function of the “meta” package.

P-values are either retrieved from single study models or a result of meta-analytic pooled estimates.

**e-Table 7. Predictors for the outcome of duration of mechanical ventilation.** Linear regression analysis across the studies

| **Variable** | **Pooled beta** | **95% Confidence Interval** | **p-value** | **Number of pooled estimates** |
| --- | --- | --- | --- | --- |
| Age | -0.21 | [-0.45 – 0.03] | 0.0833 | 2 |
| Weight | -0.04 | [-0.23 – 0.15] | 0.6737 | 1 |
| Simplified Acute Physiology Score II (SAPS II) | 0.12 | [-0.04 – 0.28] | 0.1321 | 1 |
| Injury Severity Score | 1.01 | [-0.26 – 2.67] | 0.2352 | 3 |
| Timing of admission to a specialized SCI center (ref= late) | -946.7 | [-1413.6 – -479.7] | **<0.001** | 1 |
| C1-C4 SCI level (ref= C5-C8) | 588.7 | [142.2 – 1035.2] | **0.010** | 1 |
| Thoracic Abbreviated Injury Scale (AIS) | 0.14 | [-0.01 – 0.29] | 0.0717 | 1 |
| Complete injury | 1.61 | [0 – 3.11] | **0.048** | 1 |
| Tidal volume first 24 h | -0.22 | [-0.38 – -0.05] | **0.0094** | 1 |
| Positive end-expiratory pressure first 24 h | 0.25 | [0.09 – 0.41] | **0.0027** | 1 |
| Tracheostomy | 0.83 | [0.48 – 1.18] | **<0.0001** | 1 |
| Early tracheostomy (ref= late) | -4.83 | [-17.5 – 7.8] | 0.4532 | 2 |

Corresponding beta coefficients were pooled by weighting according to the inverse variance as implemented by the “metagen” function of the “meta” package.

P-values are either retrieved from single study models or a result of meta-analytic pooled estimates.

**e-Table 8. Heterogeneity among all studies and in each subgroup (ICU and rehabilitation)**

| **Variable** | **Cochran’s Q** | **Degrees of freedom (df.Q)** | **p-value for Cochran's Q** | **I^2^** | **I^2^ ICU** | **I^2^ Rehab** | **Estimate range**  **(ICU: means)** | **Estimate range**  **(Rehab: means)** |
| --- | --- | --- | --- | --- | --- | --- | --- | --- |
| Age (years) | 1403.45 | 31 | <0.0001 | 97.8% | 98.1% | 96.7% | 26 - 72.8 | 27.1 – 57.2 |
| Gender (male) | 73.90 | 34 | 0.0001 | 54.0% | 44.1% | 0.0% | 69.2% - 100% | 76.9% - 92.9% |
| Respiratory comorbidities | 22.62 | 8 | 0.0039 | 64.6% | 67.0% | 73.0% | 0.0% - 10.5% | 4.2% - 24.1% |
| Cardiovascular comorbidities | 201.83 | 6 | <0.0001 | 97.0% | 97.9% | 47.7% | 2.4% - 30.1% | 4.2% - 17.2% |
| Other comorbidities | 317.31 | 7 | <0.0001 | 97.8% | 92.9% | 88.8% | 7.9% - 59.5% | 4.2% - 48.7% |
| Glasgow Coma Scale on admission | 228.77 | 9 | <0.0001 | 96.1% | 96.1% | NA | 9.9 – 13.7 | NA |
| Time from injury to admission | 388.77 | 13 | <0.0001 | 96.7% | 0.0% | 94.7% | 6.0 – 8.3 (hours) | 9 –378 (days) |
| Blunt as primary injury type | 727.92 | 8 | <0.0001 | 98.9% | 98.9% | NA | 5.3% – 97.3% | NA |
| Injury mechanism: fall | 111.88 | 13 | <0.0001 | 88.4% | 92.1% | 0.0% | 19.7% – 83.3% | 22.2% – 46.2% |
| Injury mechanism: transports | 115.24 | 13 | <0.0001 | 88.7% | 92.8% | 0.0% | 8.3% - 72.7% | 30.8% - 50.7% |
| Injury mechanism: assault | 30.58 | 7 | 0.0001 | 77.1% | 0.0% | 27.9% | 0.0% - 1.3% | 0.0% - 25.0% |
| Injury mechanism: sports | 16.13 | 7 | 0.0240 | 56.6% | 0.0% | 0.0% | 0.0% - 24.9% | 4.2% - 20.0% |
| Injury mechanism: other | 19.68 | 11 | 0.0499 | 44.1% | 69.1% | 0.0% | 1.5% - 14.3% | 0.0% - 7.7% |
| C3 or above | 399.06 | 26 | <0.0001 | 93.5% | 94.8% | 87.7% | 0.0% - 81.0% | 33.0% - 100% |
| C4 or above | 594.96 | 26 | <0.0001 | 95.6% | 96.7% | 87.7% | 19.0% - 100% | 0.0% - 67.0% |
| Complete lesion | 932.36 | 16 | <0.0001 | 98.3% | 98.4% | 74.5% | 17.5% - 100% | 50.7% - 83.3% |
| Extra spine lesions | 297.63 | 13 | <0.0001 | 95.6% | 95.6% | NA | 0.0% – 81.7% | NA |
| Injury Severity Score | 4735.12 | 12 | <0.0001 | 99.7% | 99.7% | NA | 17.7 –35.3 | NA |
| Weaning success | 230.52 | 23 | <0.0001 | 90% | 92.3% | 88.1% | 16.0% - 97.3% | 7.1% - 100% |
| Partial weaning | 90.50 | 6 | <0.0001 | 93.4% | NA | 93.4% | NA | 4.2% – 91.7% |
| Partial or total weaning | 74.96 | 13 | <0.0001 | 82.7% | NA | 82.7% | NA | 41.2% – 100% |
| Tracheostomy | 647.49 | 26 | <0.0001 | 96.0% | 97.1% | 0.0% | 20.6% - 100% | 88.2% - 100% |
| Decannulation | 63.59 | 9 | <0.0001 | 85.8% | 91.9% | 64.9% | 11.0% - 62.1% | 25.0% - 100% |
| Duration of mechanical ventilation | 1510.67 | 21 | <0.0001 | 98.6% | 99.0% | 95.3% | 7.7 – 75 | 7.7 – 137 |
| Total duration of mechanical ventilation + time to rehabilitation (Rehab only) | 195.37 | 7 | <0.0001 | 96.4% | NA | 96.4% | NA | 31.3 - 194.4 |
| ICU/Rehab Length of Stay | 5982.48 | 21 | <0.0001 | 99.6% | 99.7% | 56.2% | 7.5 – 46.1 | 67.8 - 99.5 |
| Hospital Length of Stay | 578.43 | 12 | <0.0001 | 97.9% | 97.9% | NA | 15.5 – 344.4 | NA |
| Pneumonia | 267.91 | 20 | <0.0001 | 92.5% | 92.3% | 90.9% | 4.5% - 73.5% | 7.1% - 95.8% |
| Mortality | 247.85 | 24 | <0.0001 | 90.3% | 92.3% | 0.0% | 0.0% - 31.8% | 0.0% - 14.3% |

Abbreviations: ICU= intensive care unit.

**REFERENCES**

1. Hozo SP, Djulbegovic B, Hozo I: Estimating the mean and variance from the median, range, and the size of a sample. *BMC Med Res Methodol* 2005; 5:1–10

2. Wan X, Wang W, Liu J, et al.: Estimating the sample mean and standard deviation from the sample size, median, range and/or interquartile range. *BMC Med Res Methodol* 2014; 14:1–13

3. Higgins J, Li T, Deeks JJ: Chosing effect measures and computing estimates of effect. In: Cochrane Handbook for Systematic Reviews of Interventions. 2020. p. 1–58.

4. Balduzzi S, Rücker G, Schwarzer G: How to perform a meta-analysis with R: A practical tutorial. *Evid Based Ment Health* 2019; 22:153–160

5. Borenstein M, Hedges L V., Higgins JPT, et al.: A basic introduction to fixed-effect and random-effects models for meta-analysis. *Res Synth Methods* 2010; 1:97–111

6. DerSimonian R, Laird N: Meta-analysis in clinical trials revisited [Internet]. *Contemp Clin Trials* 2015; 45:139–145Available from: http://dx.doi.org/10.1016/j.cct.2015.09.002

7. Clopper CJ, Pearson E. S.: Biometrika Trust The Use of Confidence or Fiducial Limits Illustrated in the Case of the Binomial Author ( s ): C . J . Clopper and E . S . Pearson Published by : Oxford University Press on behalf of Biometrika Trust Stable URL : http://www.jstor.org/stab. *Biometrika* 1934; 26:404–413

8. Higgins JPT, Thompson SG, Deeks JJ, et al.: Measuring inconsistency in meta-analyses. *Br Med J* 2003; 327:557–560

9. Kirkham JJ, Riley RD, Williamson PR: A multivariate meta-analysis approach for reducing the impact of outcome reporting bias in systematic reviews. *Stat Med* 2012; 31:2179–2195

10. Branco BC, Plurad D, Green DJ, et al.: Incidence and clinical predictors for tracheostomy after cervical spinal cord injury: A national trauma databank review. *J Trauma - Inj Infect Crit Care* 2011; 70:111–115

11. Anand T, Hanna K, Kulvatunyou N, et al.: Time to tracheostomy impacts overall outcomes in patients with cervical spinal cord injury. *J Trauma Acute Care Surg* 2020; 89:358–364

12. Call MS, Kutcher ME, Izenberg RA, et al.: Spinal Cord Injury: Outcomes of Ventilatory Weaning and Extubation. *J Trauma Inj Infect Crit Care* 2011; 71:1673–1679

13. Chiodo AE, Scelza W, Forchheimer M: Predictors of Ventilator Weaning in Individuals With High Cervical Spinal Cord Injury. *J Spinal Cord Med* 2008; 31:72–77

14. McCully BH, Fabricant L, Geraci T, et al.: Complete cervical spinal cord injury above C6 predicts the need for tracheostomy. *Am J Surg* 2014; 207:664–669

15. Jones TS, Burlew CC, Johnson JL, et al.: Predictors of the necessity for early tracheostomy in patients with acute cervical spinal cord injury: A 15-year experience [Internet]. *Am J Surg* 2015; 209:363–368Available from: http://dx.doi.org/10.1016/j.amjsurg.2014.07.016

16. Fenton JJ, Warner ML, Lammertse D, et al.: A comparison of high vs standard tidal volumes in ventilator weaning for individuals with sub-acute spinal cord injuries: A site-specific randomized clinical trial [Internet]. *Spinal Cord* 2016; 54:234–238Available from: http://dx.doi.org/10.1038/sc.2015.145

17. Kornblith L, Kutcher M, Callcut R, et al.: Mechanical Ventilation Weaning And Extubation After Spinal Cord Injury: A Western Trauma Association Multuicenter Study. *J Trauma Acute Care Surg* 2013; 75:1–20

18. Flanagan CD, Childs BR, Moore TA, et al.: Early tracheostomy in patients with traumatic cervical spinal cord injury appears safe and may improve outcomes. *Spine (Phila Pa 1976)* 2018; 43:1110–1116

19. Alizo G, Sciarretta JD, Gibson S, et al.: Multidisciplinary team approach to traumatic spinal cord injuries: a single institution’s quality improvement project. *Eur J Trauma Emerg Surg* 2018; 44:245–250

20. R Core Team: R: A language and environment for statistical computing [Internet]. *R Found Stat Comput* 2020; Available from: https://www.r-project.org/
