# Supplementary figures and images for: Separation from mechanical ventilation and survival after spinal cord injury: a systematic review and meta-analysis
Source: Ann Intensive Care. 2021 Oct 24;11:149. doi: 10.1186/s13613-021-00938-x (PMC8542415; doi:10.1186/s13613-021-00938-x)

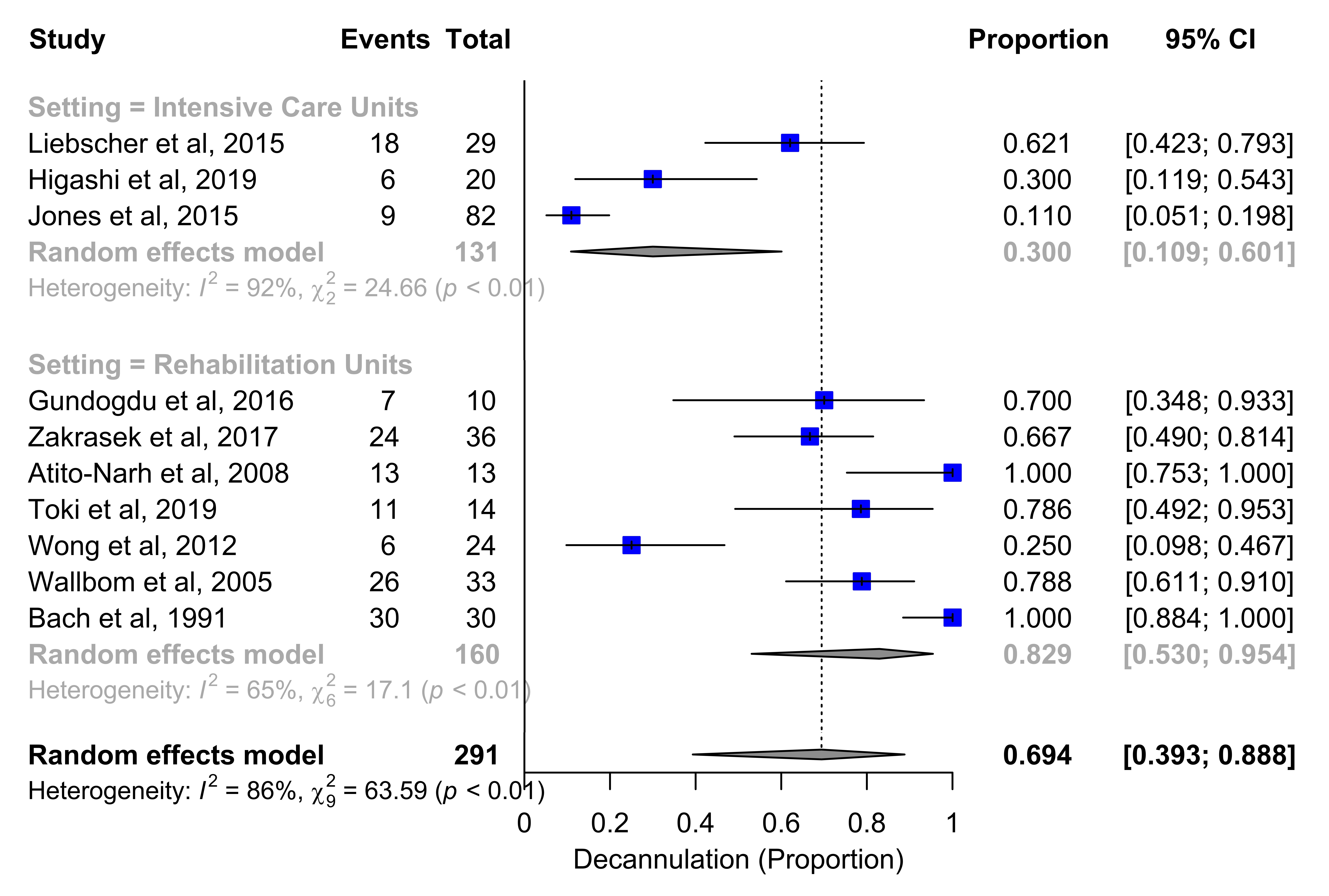

Supplement: Supplementary file 3 — Additional file 3: Figure S2. Forest plot for the probability of decannulation after tracheostomy. Studies are presented according to setting classification (Intensive Care Units vs Rehabilitation Units): both overall and subgroup estimates are reported. CI = confidence interval. [file 13613_2021_938_MOESM3_ESM.tif]

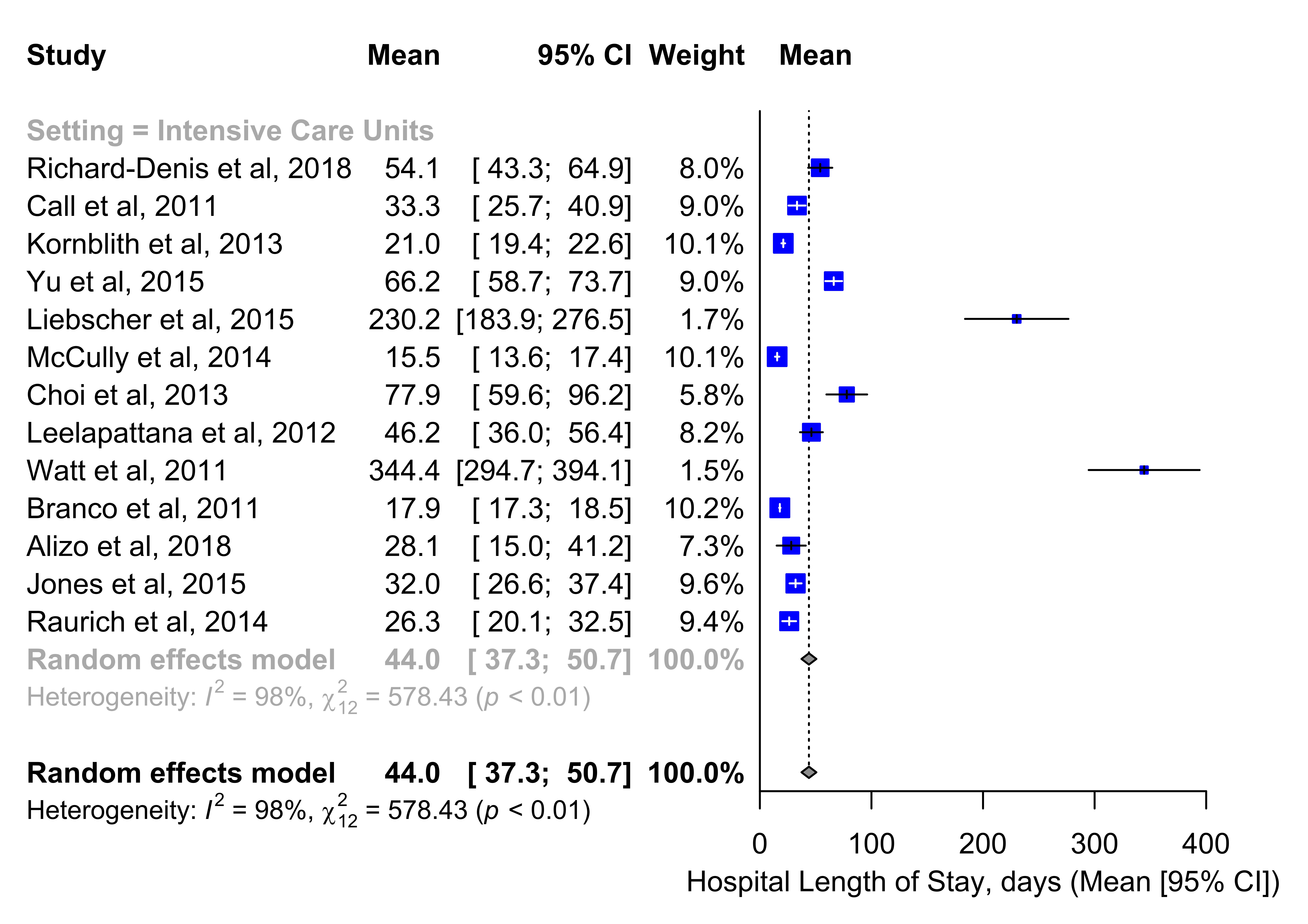

Supplement: Supplementary file 5 — Additional file 5: Figure S4. Forest plot for hospital length of stay in Intensive Care Unit setting (no data are available for Rehabilitation Units). Weight refers to the relative contribution of each study to the meta-analytic estimate and is generated using the inverse variance method. CI = confidence interval. [file 13613_2021_938_MOESM5_ESM.tif]

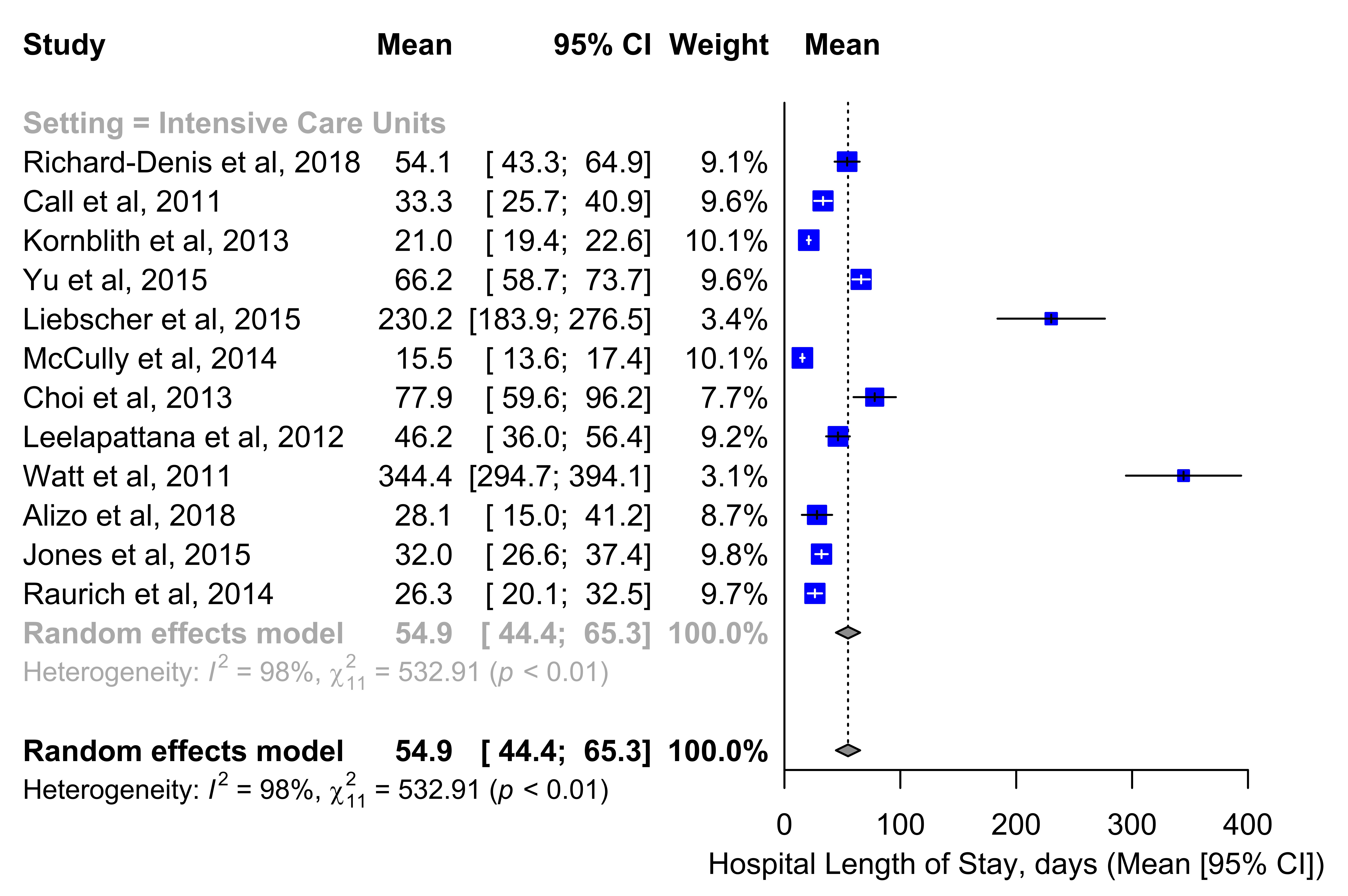

Supplement: Supplementary file 10 — Additional file 10: Figure S9. Sensitivity analysis: Forest plot for hospital length of stay excluding the two largest studies (Branco et al. [32] and Anand et al. [35]). CI = confidence interval. [file 13613_2021_938_MOESM10_ESM.tif]
